# Dynamical flexible inference of nonlinear latent factors and structures in neural population activity

**DOI:** 10.1038/s41551-023-01106-1

**Published:** 2023-12-11

**Authors:** Hamidreza Abbaspourazad, Eray Erturk, Bijan Pesaran, Maryam M. Shanechi

**Affiliations:** 1 Ming Hsieh Department of Electrical and Computer Engineering, Viterbi School of Engineering, University of Southern California, Los Angeles, CA USA; 2 Departments of Neurosurgery, Neuroscience, and Bioengineering, University of Pennsylvania, Philadelphia, PA USA; 3 Thomas Lord Department of Computer Science, Alfred E. Mann Department of Biomedical Engineering, Neuroscience Graduate Program, University of Southern California, Los Angeles, CA USA

**Keywords:** Computational neuroscience, Biomedical engineering, Computational science, Motor control

## Abstract

Modelling the spatiotemporal dynamics in the activity of neural populations while also enabling their flexible inference is hindered by the complexity and noisiness of neural observations. Here we show that the lower-dimensional nonlinear latent factors and latent structures can be computationally modelled in a manner that allows for flexible inference causally, non-causally and in the presence of missing neural observations. To enable flexible inference, we developed a neural network that separates the model into jointly trained manifold and dynamic latent factors such that nonlinearity is captured through the manifold factors and the dynamics can be modelled in tractable linear form on this nonlinear manifold. We show that the model, which we named ‘DFINE’ (for ‘dynamical flexible inference for nonlinear embeddings’) achieves flexible inference in simulations of nonlinear dynamics and across neural datasets representing a diversity of brain regions and behaviours. Compared with earlier neural-network models, DFINE enables flexible inference, better predicts neural activity and behaviour, and better captures the latent neural manifold structure. DFINE may advance the development of neurotechnology and investigations in neuroscience.

## Abstract (web summary)

Nonlinear latent factors and latent structures in the activity of neural populations can be computationally modelled to enable flexible inference and to better predict neural activity and behaviour.

## Main

Neural population activity exhibits rich spatiotemporal dynamical patterns that underlie our behaviours and functions^1–16^. Developing precise data-driven models of these complex dynamical patterns is critical both to study the neural basis of behaviour and to develop advanced neurotechnology for decoding and modulation of brain states^17,18^. Given the spatiotemporal correlations in population activity, how this activity evolves in time can be modelled more efficiently in terms of lower-dimensional latent factors. These factors can lead to scientific discovery by revealing new low-dimensional structures in coordinated population activity, which are not directly evident from the high-dimensional activity itself or from single-unit activities^1–23^. These latent factors can also decode behaviour to enable enhanced neurotechnology and brain–machine interfaces (BMIs)^17,23^. However, to enable incorporating latent factor models across broad applications such as BMIs, it is critical to develop dynamical models that not only are accurate by characterizing potential nonlinearities, but also allow for flexible inference of latent factors to seamlessly extract them. Despite much progress, this objective of simultaneously enabling both accurate nonlinear dynamical modelling and flexible inference has remained elusive.

First, to be accurate, a dynamical model should (1) capture potential nonlinearities in neural population activity and (2) have a data-efficient architecture for generalizable data-driven training. Second, to enable flexible inference, such a model should (3) be capable of both causal/real-time and non-causal inference simultaneously, and (4) allow for inference in the presence of missing neural measurements, which commonly happen in wireless neural interfaces due to wireless link interruptions^24–31^. Flexible inference is essential for developing neurotechnology and advanced implantable and/or wireless neural interfaces such as BMIs or closed-loop deep brain stimulation (DBS) systems. Flexible inference is also important for neuroscience investigations that involve causal validations^32^ or closed-loop perturbations^17,22,32–34^, long-term monitoring of brain states such as mood, or behaviours in unconstrained environments^24,27,30,35–43^ (see Discussion). If achieved, flexible nonlinear inference will enable the same trained model not only to operate causally and recursively in real-time, but also to leverage the entire length of data non-causally for more accurate inference; this ability is important for causal validation of a model of population dynamics, or for model-based closed-loop control of neural states with neurostimulation, for example, in mental disorders^17,22,33^ (see Discussion). Flexible inference will also make the model robust to missing or noisy neural measurements that happen commonly in wireless neural interfaces^24–31^. Current dynamical models of neural population activity do not satisfy all the above four properties simultaneously.

Many of these models are linear or generalized linear, often in the form of linear dynamical models (LDMs)^1,3,12,13,21,22,43–47^, and are used to infer low-dimensional latent factors^1,12,13,21^^,43–47^ or build BMIs^3^. However, while LDMs are data-efficient to train and allow for flexible inference with Kalman filtering^48^, they cannot capture the potential nonlinearities in neural population activity to describe it more accurately. Beyond linear models, recent studies have leveraged the richness of deep learning to develop generative dynamical models of neural population activity^5,14,49–52^. However, while these models can capture nonlinearity, they do not meet all the flexible inference properties outlined above. Because the inference for these models is not solvable analytically unlike LDMs, they need to empirically train an inference or recognition network simultaneously with their generative network, usually requiring the entire length of data over a trial or data segment. Thus, their inference depends on how the specific inference network is structured and is not flexible to satisfy all the above properties. Indeed, in previous generative models, including sequential autoencoders^5^ (SAEs) or LDMs with nonlinear embeddings^50^, inference is non-causal, and real-time and/or recursive inference is not directly addressed^5,14,49,50,52^ (see Discussion). Further, in these models, inference in the presence of missing observations is not directly addressed^5,14,50–52^, and using zeros instead of missing observations^53,54^ can yield suboptimal performance by changing the inherent values of missing observations during inference^55,56^ (see Results and Discussion). Similar to these generative models, predictive dynamical models of neural activity that use forward recurrent neural networks^57–59^ (RNNs) also do not enable the flexible inference properties above. While these models can perform causal inference, they do not allow for non-causal inference to leverage all data, and they do not directly address inference with missing neural observations, similar to the above generative models.

Here we report the development of a neural-network model that encompasses both flexible inference and accurate nonlinear description of neural population activity. We do so via a network architecture consisting of two sets of latent factors rather than one. One set, termed dynamic factors, captures how neural population activity evolves over a low-dimensional nonlinear manifold and approximates this evolution as linear. The other set, termed manifold factors, characterizes how this nonlinear manifold is embedded in the high-dimensional neural activity space. By separating these two sets of factors while training them together end-to-end, we can capture the nonlinearity in the manifold factors while keeping the dynamics on the manifold linear, thus enabling flexible inference by exploiting the Kalman filter on the nonlinear manifold (see Methods and Discussion). Further, using this flexible inference, we train the network to optimize the future-step-ahead prediction of neural population activity (Methods). We term this method dynamical flexible inference for nonlinear embeddings (DFINE). We emphasize that DFINE models the temporal dynamics as linear on top of the nonlinear manifold layer but not in the space of neural population activity. Indeed, we show using nonlinear simulations with the Lorenz attractor system that DFINE can still capture nonlinear dynamics well through the combination and joint training of the nonlinear manifold and the linear dynamics on this nonlinearity (Discussion). DFINE can find the best nonlinear latent manifold/embedding over which dynamics can be approximated as linearly as possible because the dynamics and manifold are trained together, rather than separately, to optimize the future-step-ahead prediction of neural population activity.

We validated DFINE in nonlinear simulations with multiple manifolds and with the stochastic Lorenz attractor system, and then compared it to benchmark linear LDM and nonlinear SAE methods across diverse behavioural tasks, brain regions and neural signal modalities. We found that DFINE not only enabled flexible inference capabilities in nonlinear modelling, but also performed significantly more accurately than both linear and nonlinear benchmarks on these neural datasets. First, given its flexible inference, DFINE robustly compensated for missing observations and seamlessly performed both causal and non-causal inference of latent factors. Second, compared to the linear and nonlinear benchmarks, DFINE significantly improved accuracy in predicting neural activity and behaviour, and in recovering the low-dimensional nonlinear neural manifold in single trials. Finally, we extended DFINE to supervise the model training with behaviour data simultaneously, such that the extracted latent factors from neural activity are more predictive of behaviour. DFINE enables the new capability of flexible inference in nonlinear neural-network modelling and enhances neural description, which are critical especially for neurotechnology and closed-loop neuroscience applications.

## Results

**Fig. 1:**
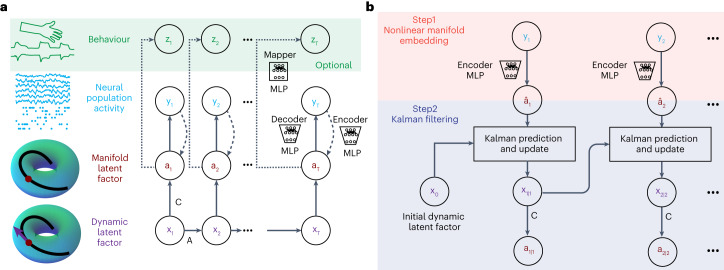
DFINE graphical model and its flexible inference method. **a**, The DFINE model with two sets of latent factors is shown. These sets consist of the dynamic and manifold latent factors, respectively, which are separated to enable flexible inference but trained/learned jointly end-to-end. The relationship between the manifold latent factors and neural observations is modelled with an autoencoder with MLP encoder and decoder networks, where the manifold latent factor is the bottleneck representation. The dashed line from neural observations to the manifold latent factor is only used for inference and is not part of the generative model. The dynamic and manifold latent factors together form an LDM, with the manifold factors being noisy observations of the dynamic factors, which constitute the LDM states. The temporal evolution of the dynamic latent factors is described with a linear dynamic equation. DFINE’s training is unsupervised by default. All model parameters (LDM, autoencoder) are learned jointly in a single optimization by minimizing the prediction error of future neural observations from their past. After this unsupervised training of the DFINE model, we use a mapper MLP network to learn the mapping between the manifold latent factors and behaviour variables. We also extend to supervised DFINE where a mapper MLP network is simultaneously trained with all other model parameters in a single optimization that now minimizes both neural and behaviour prediction errors (Methods). **b**, The inference procedure with DFINE is shown. We first get a noisy estimate of manifold latent factors using the nonlinear manifold embedding at every timepoint. With the aid of the dynamic equation, we use Kalman filtering to infer the dynamic latent factors ${{\bf{x}}}_{{t|k}}$ and refine our estimate of the manifold latent factors ${{\bf{a}}}_{{t|k}}$, with subscript *t*|*k* denoting inference at time *t* from all neural observations up to time *k*, ${{\bf{y}}}_{1:k}$. In addition to real-time filtering which is displayed (*t*|*t*), DFINE can also do smoothing. DFINE can also perform filtering and smoothing in the presence of missing neural observations (Methods). The inference method is the same for DFINE and its supervised version, and is done purely on the basis of neural observations (Methods).

We developed DFINE as a dynamical neural-network model of neural population activity with the ability to perform flexible nonlinear inference. DFINE jointly learns the nonlinear latent manifold structure in neural data and a linear model of temporal dynamics on this nonlinearity. We also devised the associated learning and inference methods for DFINE. To model neural population activity, we defined two sets of latent factors: the dynamic latent factors which characterize the linear temporal dynamics on a nonlinear manifold, and the manifold latent factors which describe this low-dimensional nonlinear manifold that is embedded in the high-dimensional neural population activity space (Fig. 1a). This separation allows the model to capture nonlinearity with the link between the manifold factors and neural population activity, while keeping the dynamics on the manifold linear, which is a design choice to enable flexible inference (Discussion). As such, these two separate sets of latent factors together enable all the above flexible inference properties by allowing for Kalman filtering on the manifold while also capturing nonlinearity via the manifold embedding. This flexible inference includes the ability to perform both causal (filtering) and non-causal (smoothing) inference, and to perform inference in the presence of missing observations. Further, inference is done recursively, such that the current inferred factor can be used to get the next inferred factor without the need to reprocess the neural data, thus enabling computational efficiency and real-time implementation (Results and Supplementary Table 1). Finally, inference can be done directly on continuous neural data streams (Methods and Discussion).

The manifold latent factors are taken as a lower-dimensional representation of the neural population activity, and the mapping between the two is characterized with an autoencoder whose decoder and encoder networks are modelled by multilayer perceptrons (MLP) (Fig. 1a). We use MLPs to model nonlinearities because they are universal approximators of any nonlinear function under mild conditions^60^. Having captured the embedding nonlinearity with the autoencoder, we now enable the model to have flexible inference properties by having the dynamic and manifold latent factors form an LDM, which is learned together with the manifold embedding (Fig. 1a). In this LDM, the manifold latent factors are noisy observations from the dynamic latent factors that constitute the LDM states and whose time evolution is described through a linear dynamic model with additive noise (Fig. 1a). Using back propagation, we jointly learn all the model parameters by minimizing the prediction error of future neural observations from past neural observations (future-step-ahead prediction error), measured using the mean-squared error (MSE). Since both dynamic and manifold latent factors are learned together in an end-to-end gradient-descent optimization, DFINE learns the best nonlinear manifold over which dynamics can be approximated as linearly as possible (Discussion).

DFINE’s model training is unsupervised by default. For situations when specific behavioural variables are of interest and available during training, we extend DFINE to supervised DFINE so that learning of the model is informed by how predictive the learned manifold latent factors would be not only of future neural observations but also of behavioural variables. This is done during training by introducing an extra link from the manifold latent factors to continuous behaviour variables (Fig. 1a), termed the mapper network modelled with an MLP, and by modifying the cost function to simultaneously include both behaviour and neural prediction errors (Methods). This additional link is purely added during training and removed afterwards during inference on test data. This leads to a learned model that is identical to the original model in terms of architecture and inference but just with different parameter values. Importantly, inference is again done purely from neural observations (Fig. 1b and Methods). Unless otherwise stated, all DFINE results are with unsupervised training.

In addition to showing that DFINE enables the new capability of combining neural-network modelling with flexible inference for neural population activity, we also compare it to benchmarks of linear LDM and nonlinear SAE. To show the generalizability of DFINE across behavioural tasks, brain regions and neural signal modalities, we perform our analyses across multiple independent datasets. For SAE, we use the architecture named latent factor analysis via dynamical systems or LFADS^5^, which is a common benchmark nonlinear model of neural population activity^13,49,52^. For each dataset and algorithm, we infer the latent factors from the trained models. The latent factors correspond to the manifold latent factors in DFINE (Methods), to the state in the state–space model in LDM^21^, and to the dynamic factors (the representation layer right after the generator RNN) in SAE^5^. Unless otherwise stated, we use smoothing to infer the latent factors for all methods for consistency in comparisons. We report all the quantifications using 5-fold cross-validation, where the values are calculated in the held-out test set (Methods). After extracting the latent factors, in the training set, we learn classification or regression models for discrete or continuous behavioural variables, respectively.

We quantify the cross-validated behaviour prediction accuracy with area under the curve (AUC) of the receiver operating characteristic^61^ for discrete classifiers and with Pearson’s correlation coefficient (CC) for continuous regressions. The neural prediction accuracy is calculated with one-step-ahead prediction accuracy, the accuracy of predicting neural observations one step into the future from their past (Methods). We also evaluate the neural reconstruction accuracy, defined as how well inferred latent factors, whether via causal filtering or via non-causal smoothing, reconstruct the current neural observations. Error values are computed in normalized root MSE (NRMSE) defined as root MSE normalized by the variance of the ground-truth observations to allow pooling of the values across sessions given scaling differences (Methods). To assess how well the structure of the manifold is revealed in single trials, we apply topological data analysis^62^ (TDA) on the extracted latent factor trajectories in test sets (Methods).

### Neural recordings and experimental tasks

We demonstrate the DFINE method using both extensive numerical simulations with multiple nonlinear manifolds and with the stochastic Lorenz attractor system, as well as four diverse datasets containing distinct behavioural tasks, brain regions and neural signal modalities to show the generalizability of the method. We model two neural modalities that are commonly used, which are smoothed neuronal firing rates and local field potentials (LFP). These datasets contain four behavioural tasks, and three of the four datasets have unit-based recordings with a relatively high number of tuned channels (at least 40 per session)^12,63–66^. The details of these four datasets are as follows.

**Fig. 2:**
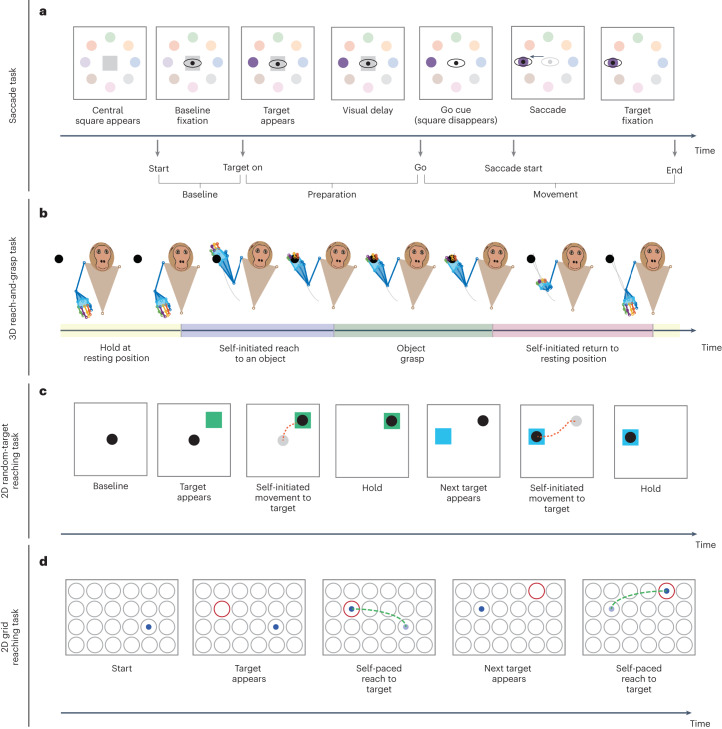
Experimental tasks. **a**, Events for the saccade task are shown. At the beginning of each trial, the subject is required to fixate its eyes on a baseline location (grey square). After fixation, the target is illuminated on the screen (Target On event). After a visual delay, the fixation square disappears, which signals a go command (Go event). Then the subject performs the saccade (Saccade Start event) and holds fixation on target to receive a liquid reward (End event). We define the preparation and movement periods as the duration between Target On and Go events, and the duration between Go and End events, respectively. **b**, In the naturalistic 3D reach-and-grasp task, an experimenter continuously moves a wand to diverse locations within a 3D area in front of the subject. The subject naturalistically reaches to the object on the wand, grasps it and returns its arm to the resting position. Movements are self-initiated without a specific go cue or timing instructions to isolate any parts of movements. **c**, In the 2D random-target reaching task, the subject controls a cursor (black circle) using a manipulandum. The task consists of several sections, with each section having 4 sequential reaches to random targets (shown by coloured squares) that appear on the screen (only 2 reaches are shown here for simplicity). The subject performs self-initiated movements towards the targets. After a brief hold on a target, the next random target appears at a pseudo-random location on the screen. **d**, In the 2D grid reaching task, the subject controls a cursor (blue dot) on a 2D grid in a virtual-reality environment by moving its fingertip. Once the target (red circle) appears on one of the circular locations on the grid (grey circles), the subject performs a self-paced movement towards the target, after which another target appears from the set of possible targets.

In the first dataset, a macaque monkey (Monkey A) performed saccadic eye movements towards 1 of 8 peripheral targets on a screen in a visually guided oculomotor delayed response task, which we refer to as the saccade task^67^ (Fig. 2a and Methods). Each trial started with presentation of a central fixation square to which the monkey was required to maintain fixation, followed by Target On, Go, Saccade Start and End time events (Fig. 2a and Methods). We defined the preparation period as the time between Target On and Go events, and the movement period as the time between Go and End events (Fig. 2a). We processed raw LFP signals in lateral prefrontal cortex (PFC), given their importance in saccadic eye movement representation^13,68^ (Methods).

In addition, we used three independent motor datasets to show generalizability. For all motor datasets, we took the Gaussian-smoothed firing rates as the neural signal to be modelled (Methods). For one of the datasets (3D naturalistic reach-and-grasp), we also modelled the LFPs. The first motor dataset was a 3D naturalistic reach-and-grasp task, where the monkey (Monkey J) performed naturalistic reach-and-grasps towards diverse locations in 3D space, while the 3D endpoint hand position and velocity were measured and taken as the behaviour variables^12^ (Fig. 2b and Methods). The neural recordings covered primary motor cortex (M1), dorsal premotor cortex (PMd), ventral premotor cortex (PMv) and PFC. The second motor dataset was a publicly available 2D random-target reaching task^63,64^, where PMd activity was recorded while the monkey (Monkey T) made sequential 2D reaches on a screen using a cursor controlled with a manipulandum, and the 2D cursor position and velocity were tracked as the behaviour (Fig. 2c and Methods). The third motor dataset was a publicly available 2D grid reaching task^65,66^, where M1 activity was recorded while the monkey (Monkey I) controlled a cursor on a 2D surface in a virtual-reality environment via its fingertip movements whose 2D position and velocity were tracked as the behaviour (Fig. 2d and Methods).

### DFINE can learn the dynamics on diverse nonlinear manifolds and enables flexible inference in simulated datasets

**Fig. 3:**
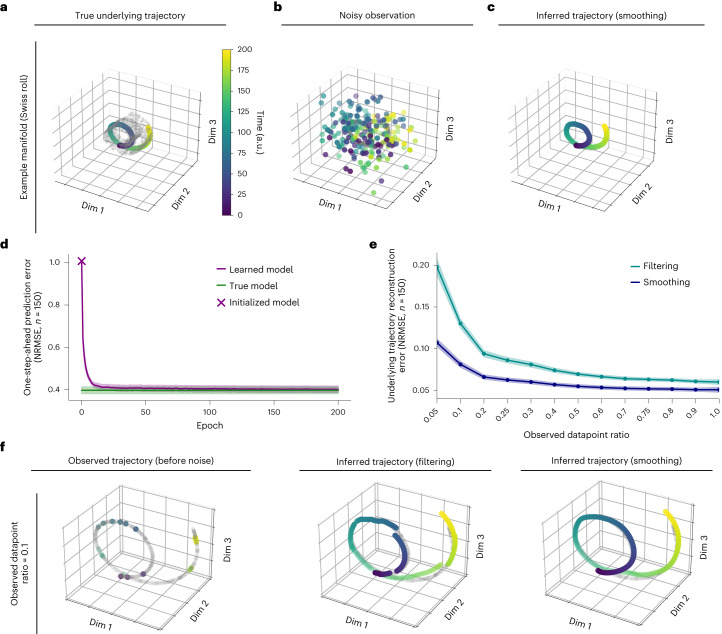
DFINE can learn the dynamics on nonlinear manifolds and enables flexible inference in the presence of missing observations in simulated datasets. **a**, A sample simulated trajectory is shown for an example manifold (Swiss roll). The grey points are samples from the underlying manifold. Colour evolution on the trajectory indicates time evolution into a trial as shown in the colour bar, which represents discrete time-steps. **b**, Noisy observations of the trajectory are shown with the same colour convention as in **a** and on top of the grey true trajectory. **c**, After learning the DFINE model, inferred trajectory with smoothing is shown, which is essentially on top of the true trajectory represented with grey dots and masking it. **d**, The learned models’ one-step-ahead prediction error converges to that of the true models. The plot shows the mean one-step-ahead prediction error for the learned and true models across all simulated sessions, cross-validation folds and trials. The shaded areas represent the 95% confidence bound of the mean. DFINE starts from a randomly initialized model, which has a chance-level one-step-ahead prediction of 1. **e**, Neural reconstruction error between the inferred and true trajectories is shown for smoothing and filtering across various observed datapoint ratios. Dots represent the mean across simulated sessions and cross-validation folds, and shaded areas represent the 95% confidence bound. Smoothing and filtering errors are essentially unchanged as samples are dropped even up to 0.2 and 0.5 observed datapoint ratios, respectively. These errors are significantly lower than chance level of 1 for all ratios (*P* < 8.7 × 10^−7^, one-sided Wilcoxon signed-rank test). Smoothing is more accurate than filtering across all ratios (*P* < 8.7 × 10^−7^, one-sided Wilcoxon signed-rank test). **f**, An example trajectory with missing observations and its smoothing and filtering inference for observed datapoint ratio of 0.1 are shown. Coloured dots in the left panel are observed datapoints (before noise is added) and the grey dots are shown to visualize the true underlying trajectory.

We first validated the DFINE model and its learning algorithm in numerical simulations. Given the plausibility of ring-like, spiral-like and toroidal structures in neural population activity in previous studies^7,11,16,69^, and to show the generality of the method across various manifold types, we simulated trajectories on Swiss roll, Torus and ring-like manifolds as a proof of concept (Fig. 3 and Methods). We synthesized 30 different simulated sessions (each with 250 trials) with randomly selected noise values and with the manifolds uniformly chosen from the above possibilities (Methods). Given the noisy nature of neural recordings, the simulation observations were taken as the noisy realizations of the trajectories on the manifold (Fig. 3b). It is not easy to directly compare the learned and the true model parameters for two reasons. First, we simulate the data using analytical nonlinear manifold equations while we use neural networks in DFINE to model them. Second, there are many similarity transforms for model parameters that describe the data statistics equivalently^59,70^. As such, we instead used one-step-ahead prediction error as a measure of convergence to the true model^70^.

We found that DFINE can correctly infer the trajectories on the manifolds both with causal filtering and with non-causal smoothing, even from noisy observations (Fig. 3a–c) and even in the presence of missing observations (Fig. 3e,f). First, the learned model’s one-step-ahead prediction error converged to that of the true model (Fig. 3d). The difference between learned and true model errors, normalized by the true model error, decreased from 1.564 ± 0.110 (mean ± s.e.m.) for randomly initialized models to 0.012 ± 0.004 for the learned model, indicating convergence (Fig. 3d). Second, the same DFINE model enabled inference both causally and non-causally, and even in the presence of missing observations (Fig. 3e,f). To show this, we randomly dropped neural observation datapoints from each trial to achieve a desired observed datapoint ratio, which is defined as the ratio of the datapoints that are maintained/not-dropped to the total number of datapoints (Methods). This random drop was done to emulate the common problem of data drop in wireless neural interfaces (Discussion). DFINE’s inferences even for ratios as low as 0.2 were similar to when all datapoints were retained, showing that DFINE could use the learned dynamics to compensate for missing observations (Fig. 3e; see dark blue curve for example). Further, even for ratios as low as 0.05, DFINE still performed better than chance level of 1 (Fig. 3e; $P < 8.7\times {10}^{-7}$, *N*_s_ = 150, one-sided Wilcoxon signed-rank test). Third, smoothing significantly improved the inference of trajectories ($P < 8.7\times {10}^{-7}$, *N*_s_ = 150, one-sided Wilcoxon signed-rank test) because it could also leverage the future neural observations, and this improvement due to smoothing was more prominent in the lower observed datapoint ratios (Fig. 3e). Indeed, smoothing could infer the trajectories even for ratios as low as 0.1 (Fig. 3f). Overall, the simulation analysis showed that DFINE can learn the dynamics on diverse nonlinear manifolds, perform flexible inference both causally (filtering) and non-causally (smoothing), and succeed even in the presence of missing observations.

**Extended Data Fig. 1:**
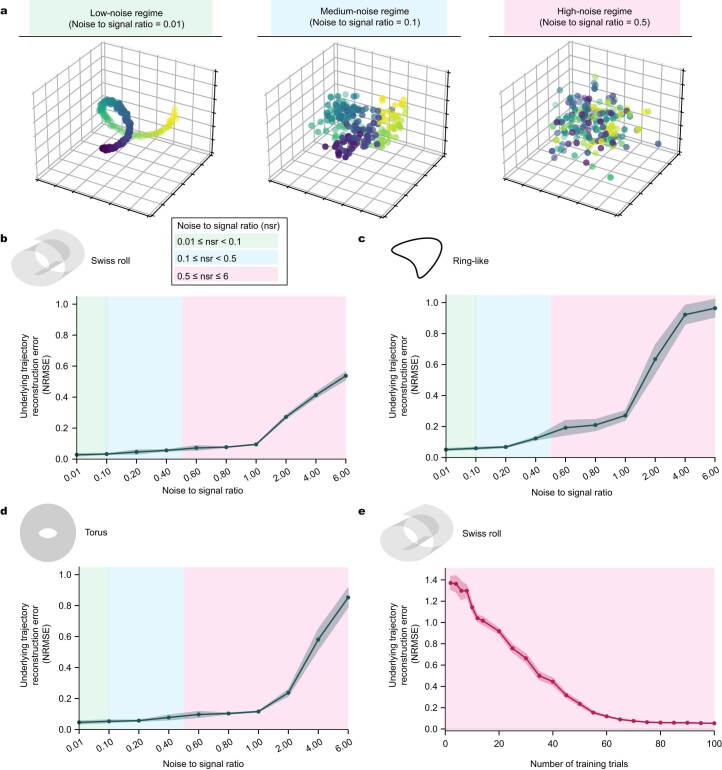
Characterization of DFINE’s performance vs. the observation noise and the amount of training data. **a**, Examples of noisy observations of the latent factor trajectory are shown in three different noise regimes. Figure convention is similar to Fig. 3b. **b**, The underlying trajectory reconstruction error is shown for the Swiss-roll manifold at various noise to signal ratios (nsr). Noise to signal ratio is calculated by dividing the standard deviation of the observation noise to that of the original signal. Each dot represents the mean reconstruction error across simulated sessions and cross-validation folds (*n*=50). The solid line represents the mean and the shaded area shows the 95% confidence bound of the mean. **c**, Similar to **b** for the ring-like manifold. **d**, Similar to **b**, for the Torus manifold. **e**, The cross-validated reconstruction error of the underlying trajectory is shown for the Swiss roll manifold at a given noise to signal ratio (0.5, corresponding to the right-most panel in **a**) vs. the number of training trials. DFINE’s performance converges when around 75 trials are available. Similar to **b-d**, each dot represents the mean reconstruction error across simulated sessions and cross-validation folds (*n*=50), the solid line represents the mean, and the shaded area shows the 95% confidence bound of the mean.

Having established DFINE’s capability in learning the dynamics on nonlinear manifolds and enabling flexible inference, we characterized how the observation noise and amount of training data affect DFINE’s performance (Extended Data Fig. 1). For each manifold type, we swept the observation noise to obtain low to high noise to signal ratios, defined as the ratio of noise standard deviation to the ground-truth signal standard deviation (Extended Data Fig. 1a–d). We found that while DFINE’s performance decreased with higher noise as expected, its performance stayed robust in a wide range of noise to signal ratios even including high ratios (Extended Data Fig. 1b–d). Also, this robustness held for all three manifolds examined here, but the range for robustness varied across manifolds, with the Swiss-roll manifold and the ring-like manifold being the most and the least robust to noise, respectively (Extended Data Fig. 1b vs c). To characterize DFINE’s performance with the available training data, we varied the number of training trials for the Swiss-roll manifold from 1 to 100 at a noise to signal value similar to that in the main simulations in Fig. 3. We observed that DFINE started to predict better than chance in the test set at around 20 training trials and converged at around 75 training trials (Extended Data Fig. 1e).

### DFINE extracts single-trial latent factors that accurately predict neural activity and behaviour

**Fig. 4:**
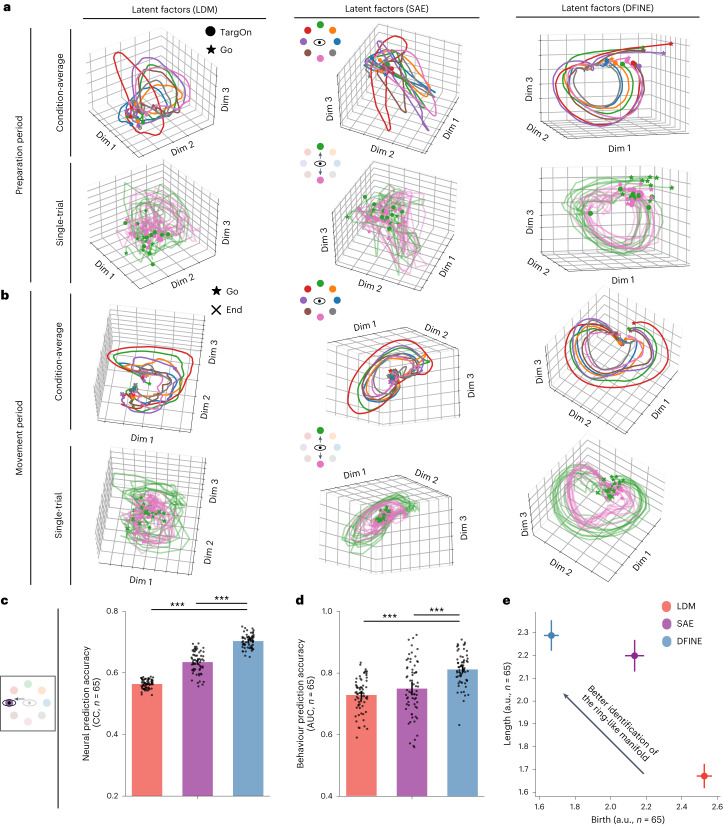
In the saccade dataset, DFINE outperformed benchmark methods in behaviour and neural prediction accuracy, and more robustly extracted the ring-like manifold in single trials. **a**, Condition-average (top row) and single-trial (bottom row) latent factor trajectories for an example session are shown for DFINE, LDM and SAE during the preparation period. Each colour represents one target, that is, condition. **b**, Similar to **a**, for the movement period. **c**, DFINE had significantly higher neural prediction accuracy than LDM and SAE. All models had 16D latent factors (see Supplementary Fig. 1 for convergence). Black dots represent the accuracy in each cross-validation fold of each session, bars represent the mean and error bars represent the 95% confidence bound. Asterisks indicate significance of comparison (***P < 0.0005, one-sided Wilcoxon signed-rank test). **d**, Behaviour prediction measured by target classification accuracy was better with DFINE than with LDM and SAE. Convention is the same as in **c**. **e**, TDA analysis on single-trial latent factors during the movement period is shown. The top left area of the plots corresponds to more robust extraction of the ring-like manifold (Methods). Circles represent mean across sessions and cross-validation folds, and error bars represent the 95% confidence bound. TDA’s most persistent 1D hole had a significantly earlier birth and lasted significantly longer for DFINE than for LDM and SAE (*P* *<* 5 × 10^−4^, one-sided Wilcoxon signed-rank test).

We then applied DFINE on the four independent datasets to show that it not only allows for flexible inference but also for accurate neural and behaviour prediction. We compared DFINE’s single-trial latent factors to those of the linear (LDM) and nonlinear (SAE) benchmarks (Methods). We first visualized the condition-average and single-trial latent factor trajectories for the saccade task during both preparation (Fig. 4a) and movement periods (Fig. 4b), where condition is defined as the saccade target. We found that the latent factors inferred with DFINE not only captured inter-condition variabilities during movements even in single trials (Fig. 4b), but also exhibited smooth trajectories with a discernible manifold structure in these noisy single trials (Fig. 4a,b). This was unlike LDM that generally had noisier single-trial latent factor trajectories, and unlike SAE whose latent factor trajectories, while smooth, captured smaller inter-condition variabilities in single trials (Fig. 4a,b).

We next quantified this ability to capture inter-condition variability by computing the single-trial neural and behaviour prediction accuracies of 16-dimensional (16D) latent factors for each method. We picked this dimension because it was sufficient for the performance of all methods to converge across all datasets (Supplementary Fig. 1; SAE’s dynamic state dimension was taken to be much higher at 64; see Discussion and Methods). Note that during a given trial or data segment, SAE does not predict the neural observations into the future from their past because it needs to use all neural observations until the end of a trial/segment to get the initial condition and then to extract the factor trajectories at every time step from this initial condition. Thus, while we computed the one-step-ahead neural prediction error for DFINE and LDM using only past neural observations, we gave SAE the advantage of doing neural reconstruction with smoothing on the basis of both past and future neural observations instead.

**Fig. 5:**
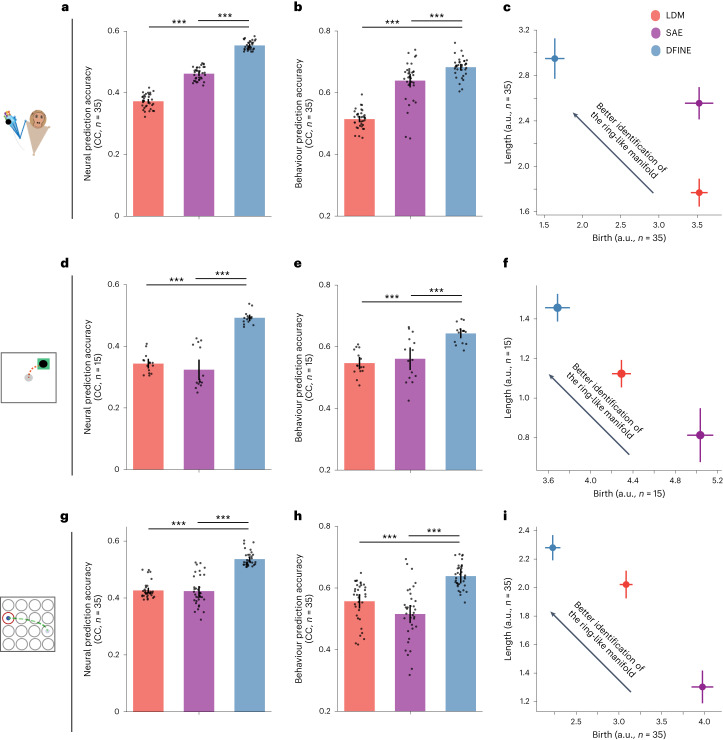
In the motor datasets, DFINE outperformed benchmark methods in behaviour and neural prediction accuracy and in robustly extracting the predictive ring-like manifold. Figure convention for bars, asterisks for significance and for the TDA plots are the same as in Fig. 4. DFINE again outperformed benchmarks in terms of neural prediction, behaviour prediction and robust extraction of the manifold structure in single trials in the naturalistic reach-and-grasp task (**a**–**c**), random-target reaching task (**d**–**f**) and 2D grid reaching task (**g**–**i**).

DFINE was better at neural prediction not only compared with LDM but also compared with SAE across all datasets (Figs. 4c and 5a,d,g). In comparison with SAE and LDM, respectively, DFINE improved the accuracy of neural prediction in the 3D naturalistic reach-and-grasp task by 19.9 ± 1.8% and 49.0 ± 3.7% (Fig. 5a; $P < 1.2\times {10}^{-7}$, *N*_s_ = 35, one-sided Wilcoxon signed-rank test), in the 2D random-target reaching task by 56.7 ± 26.7% and 43.9 ± 7.3% (Fig. 5d; $P < 3.1\times {10}^{-5}$, *N*_s_ = 15, one-sided Wilcoxon signed-rank test), in the 2D grid reaching task by 27.8 ± 6.5% and 25.9 ± 2.2% (Fig. 5g; $P < 1.2\times {10}^{-7}$, *N*_s_ = 35, one-sided Wilcoxon signed-rank test) and in the saccade task by 10.9 ± 1.7% and 24.7 ± 1.3% (Fig. 4c; $P < 1.2\times {10}^{-12}$, *N*_s_ = 65, one-sided Wilcoxon signed-rank test). Similar results held for the comparison of DFINE with SAE in terms of neural reconstruction with smoothing (Supplementary Fig. 2; see also Discussion).

In addition to its better neural prediction, DFINE also had higher behaviour prediction accuracy compared with LDM and SAE in all tasks (Methods). In the motor tasks, improvements compared with SAE and LDM, respectively, were 7.7 ± 5.7% and 33.0 ± 4.0% in the 3D naturalistic reach-and-grasp task (Fig. 5b; $P < 3.7\times {10}^{-5}$, *N*_s_ = 35, one-sided Wilcoxon signed-rank test), 16.8 ± 17.1% and 17.9 ± 3.9% in the 2D random-target reaching task (Fig. 5e; $P < 2.6\times {10}^{-4}$, *N*_s_ = 15, one-sided Wilcoxon signed-rank test) and 21.2 ± 7.5% and 11.6 ± 7.0% in the 2D grid reaching task (Fig. 5h; $P < 5.2\times {10}^{-7}$, *N*_s_ = 35, one-sided Wilcoxon signed-rank test). Also, for the saccade task, DFINE latent factors better predicted the saccade target class during the movement periods (Methods), with the saccade target classification AUC being 9.7 ± 3.9% and 11.8 ± 2.9% better than those of SAE and LDM, respectively (Fig. 4d; $P < 9.6\times {10}^{-7}$, *N*_s_ = 65, one-sided Wilcoxon signed-rank test).

**Extended Data Fig. 2:**
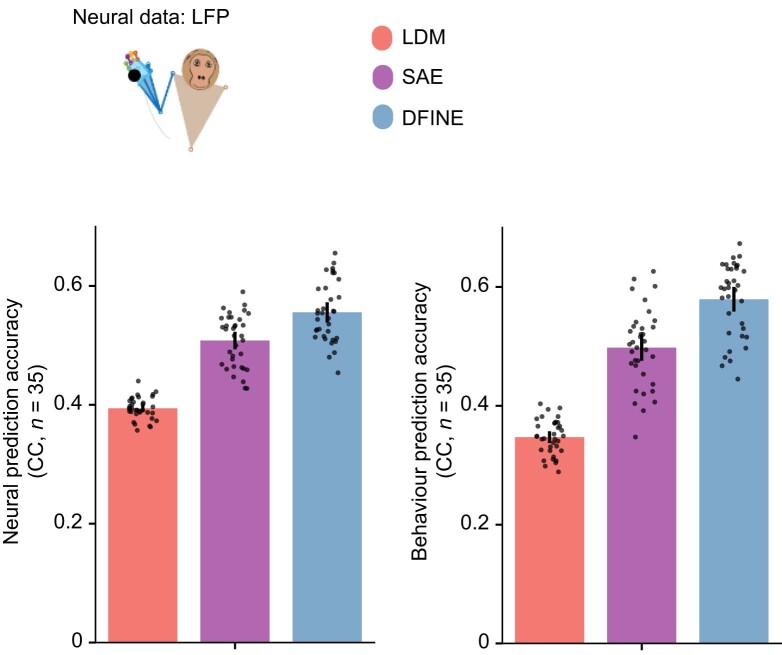
DFINE outperforms LDM and SAE in the 3D naturalistic reach-and-grasp task also when using the LFP modality instead of smoothed firing rates. Neural and behaviour prediction accuracies of DFINE versus the benchmark methods are shown in **a** and **b**, respectively. Figure convention is similar to Fig. 5. In both neural and behaviour prediction accuracies, DFINE was again better than SAE (*P* < 2.7 × 10^−5^, *N*_*s*_=35, one-sided Wilcoxon signed-rank test) while SAE was better than LDM ($P < 2.5\times {10}^{-7}$, *N*_*s*_=35, one-sided Wilcoxon signed-rank test).

To show that DFINE improvements in the motor datasets are not specific to the smoothed firing rate modality and to provide further comparative evidence, we also compared DFINE with LDM and SAE in the 3D naturalistic reach-and-grasp dataset by processing the LFP signals instead of smoothed firing rates. We observed that DFINE again had a higher neural and behaviour prediction accuracy for LFPs (Extended Data Fig. 2; $P < 2.7\times {10}^{-5}$, *N*_s_ = 35, one-sided Wilcoxon signed-rank test).

**Extended Data Fig. 3:**
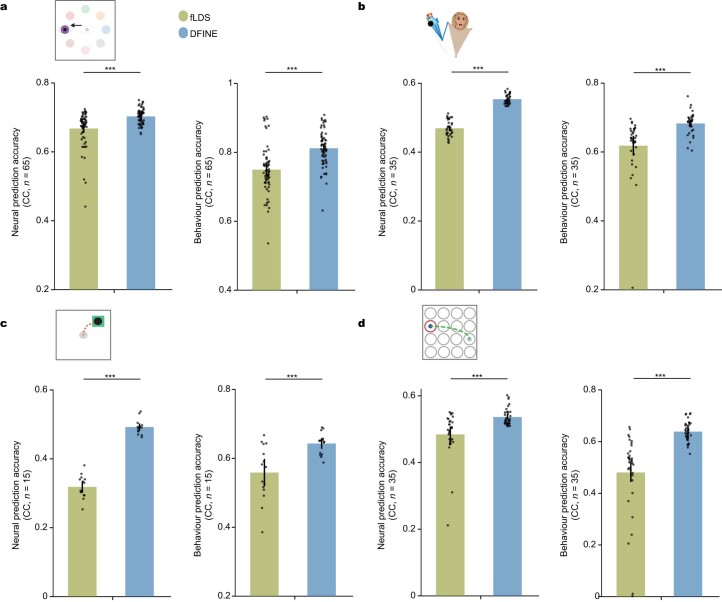
DFINE outperforms fLDS across the four experimental datasets. Neural and behaviour prediction accuracies were both significantly higher in DFINE compared with fLDS in the saccade task (**a**), the 3D naturalistic reach-and-grasp task (**b**), the 2D random-target reaching task (**c**), and the 2D grid reaching task (**d**). Figure convention is similar to Figs. 4 and 5. Beyond performance gains, as a major goal and benefit, DFINE provides the new capability for flexible inference in neural population activity unlike fLDS that performs non-causal inference and does not directly address missing data.

As another comparative evidence and to further highlight the methodological advances of DFINE, we also compared DFINE to fLDS^50^ (Methods), which is another competitive model of neural population activity that uses an LDM with a direct nonlinear observation equation. First, the new flexible inference capability that is a main goal of DFINE is not enabled with fLDS, which instead performs non-causal smoothing and does not address missing data directly (Discussion). Second, we found that even though DFINE’s inference is flexible, DFINE still had higher neural and behaviour prediction accuracies compared with fLDS across all the four experimental datasets (Extended Data Fig. 3; $P < 3.1\times {10}^{-5}$, ${N}_{\rm{s}} > 15$, one-sided Wilcoxon signed-rank test). The comparison with fLDS also shows the benefit of the methodological advances in DFINE including its extra noisy manifold factor layer, its flexible inference capability and its future-step-ahead prediction cost, which also ultimately lead to its better performance (see Discussion and Methods). Finally, DFINE’s runtimes for both learning and inference were also faster than those of the other nonlinear benchmark methods, that is, SAE and fLDS (Supplementary Table 1).

These results demonstrate that in addition to enabling flexible dynamical inference unlike earlier neural-network models of neural activity, DFINE can extract single-trial latent factors that are more predictive of both behaviour and neural activity compared with the benchmark linear and nonlinear methods.

### Simulations with the nonlinear stochastic Lorenz attractor system

**Extended Data Fig. 4:**
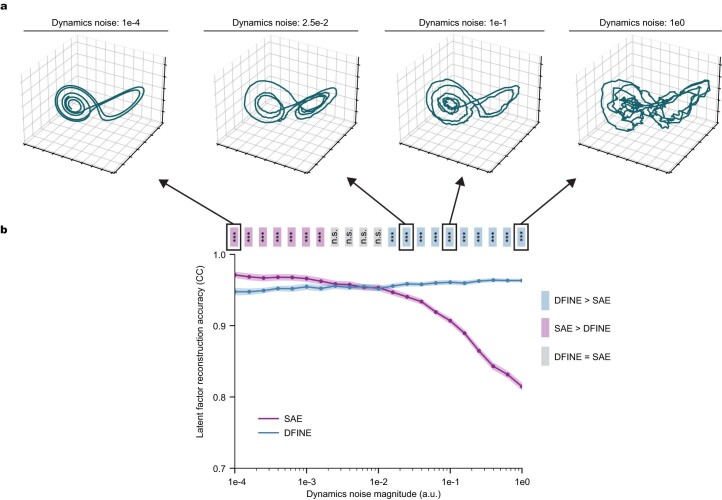
DFINE outperforms SAE in the presence of stochasticity in the nonlinear temporal dynamics of the Lorenz attractor system, while SAE outperforms DFINE when the dynamics are almost deterministic. **a**, Examples of latent factor trajectories for the stochastic Lorenz attractor system across various dynamics noise magnitudes (quantified as the noise variance) (Methods). The arrows associate example trajectories with the comparisons in **b** to provide visualization. **b**, The cross-validated reconstruction accuracies of the ground-truth Lorenz latent factors for SAE and DFINE are shown for various dynamics noise magnitudes. The solid lines are the mean latent factor reconstruction accuracy across simulated systems and cross-validation folds (*n*=100). The shaded area around the mean represents the 95% confidence bound. Figure convention for the significance asterisks is similar to Fig. 4. For nonlinear temporal dynamics that are almost deterministic, SAE outperformed DFINE (dynamics noise magnitudes smaller than ~2.5 × 10^−3^). With the increase in the dynamics noise magnitude, SAE performance degraded while DFINE performance stayed robust. DFINE significantly outperformed SAE when there was stochasticity in nonlinear temporal dynamics and specifically when the dynamics noise magnitude was larger than ~10^−2^. DFINE’s inference explicitly takes into account stochasticity by incorporating the stochastic noise variables during inference; this helps DFINE perform well and robustly in the presence of stochasticity in the Lorenz dynamics here (Methods). The *x* axis is in log scale and shows the variance of the dynamics noise (Methods).

To gain intuition as to why DFINE outperforms SAE in our data, we simulated a stochastic Lorenz attractor system, which is a well-known dynamical system with nonlinear temporal dynamics. We swept the stochasticity/noise in state dynamics in these simulations. Extended Data Fig. 4a shows examples of latent factor trajectories for the Lorenz attractor system under various stochasticity/noise regimes. After training the DFINE and SAE models in the training set, we then quantified the reconstruction accuracy of the latent factors in the test set similar to a previous work^5^ (Methods).

First, we observed that DFINE was able to capture the Lorenz nonlinear temporal dynamics through its combined learning of a nonlinear latent manifold and linear dynamics on this manifold, resulting in accurate latent trajectory reconstruction (Extended Data Fig. 4b). Second, as the stochasticity increased in nonlinear dynamics: (1) SAE performance degraded while DFINE’s performance stayed robust (Extended Data Fig. 4b) and (2) DFINE significantly outperformed SAE (Extended Data Fig. 4b when the state dynamics noise is larger than ~10^−2^). However, when the nonlinear dynamics were almost deterministic, SAE outperformed DFINE (Extended Data Fig. 4b when the state dynamics noise is less than ~$2.5\times {10}^{-3}$). These Lorenz simulations suggest that one reason why DFINE outperforms SAE on our four neural datasets can be because of inherent stochasticity in the neural dynamics, and because potential model mismatches can exist for any model of real-world data and these mismatches can also be captured as noise/stochasticity (no model is perfect for data). DFINE accounts for such stochasticity during inference by learning and then explicitly incorporating stochastic noise variables in its inference (Methods and Extended Data Fig. 4).

### DFINE more robustly extracts the manifold structure in single trials

Because the latent trajectories in DFINE were more predictive of neural activity and behaviour, we used visualization and TDA analyses on these trajectories to study the latent manifold structure in data and found these trajectories to reveal a ring-like manifold structure (Fig. 4a,b and Supplementary Fig. 3). Further, DFINE more robustly captured this ring-like nonlinear manifold structure in single-trial data compared with LDM and SAE. First, visualization of DFINE revealed a ring-like manifold structure in both condition-average and single-trial latent factors during both preparation and movement periods in the saccade task (Fig. 4a,b) and during the movement periods in the motor tasks (Supplementary Fig. 3). Also, this ring-like structure was much less apparent in single trials for LDM and SAE (for example, Fig. 4a), while it could be seen in their condition-average trajectories (for example, Fig. 4b and Supplementary Fig. 3). To quantify this observation and whether DFINE was better able to extract this manifold in single trials, we used TDA which uses persistent homology to quantify whether there exist holes in data, and if so how many^62^ (Methods). TDA finds multidimensional holes (for example, 1D hole is a ring and 2D hole is a 2D void) in data by growing the radius of ***ϵ***-balls around datapoints (Methods). If holes exist, a model that finds holes that are born earlier and last longer (that is, are more persistent) is more robust in revealing the manifold structure in single trials. Consistent with observing a ring in low-dimensional visualizations above, TDA revealed a persistent 1D hole in low-dimensional latent factors, which we then analysed. Compared with the other methods, in DFINE, the birth of TDA’s most persistent 1D hole was significantly earlier and its length was significantly larger during both preparation and movement periods for the saccade task (Fig. 4e and Supplementary Fig. 4; $P < 5\times {10}^{-4}$, one-sided Wilcoxon signed-rank test), and during movement periods for all motor tasks (Fig. 5c,f,i; $P < 5\times {10}^{-4}$, one-sided Wilcoxon signed-rank test).

**Extended Data Fig. 5:**
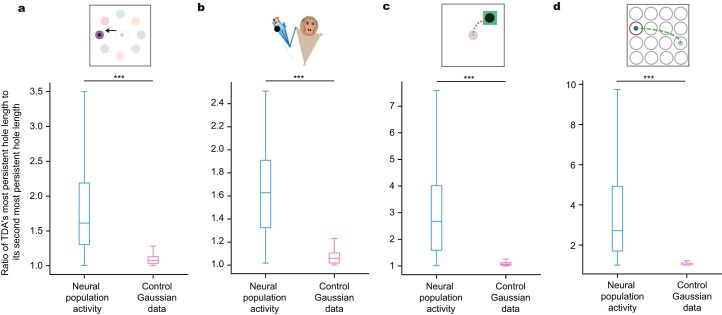
TDA analysis directly on the observed neural population activity reveals a ring-like manifold structure. The ring-like manifold was found in the saccade task (**a**), the 3D naturalistic reach-and-grasp task (**b**), the 2D random-target reaching task (**c**), and the 2D grid reaching task (**d**). We performed TDA directly on the observed neural population activity ${{\bf{y}}}_{t}\in {{\mathbb{R}}}^{{{{n}}}_{{{y}}}\times 1}$ without any modelling and quantified the ratio between the length (duration between birth and death) of the most persistent one-dimensional hole to that of the second most persistent one-dimensional hole. This ratio was significantly larger than the control data in all datasets (**a-d**), again indicating the existence of a ring-like manifold structure in the data (even without any modelling). The control data is taken as a unit-norm Gaussian noise in ${{\mathbb{R}}}^{{{{n}}}_{{{y}}}\times 1}$ because we z-score each trial’s latent factors for the TDA analysis to remove scaling differences (Methods). The line inside boxes shows the median, box edges represent the 25^th^ and 75^th^ percentiles, and whiskers show the minimum and maximum values after removing the outliers. We removed outliers that were outside the 3-standard-deviation range from the mean on each side. Figure convention for asterisks is similar to Fig. 4.

To show that the ring-like manifold structure is not an artefact of DFINE or other models, we directly performed TDA on the neural population activity without any modelling. We again observed that TDA determined a significant ring-like manifold structure across all four datasets (Extended Data Fig. 5). We emphasize that a priori, it is neither desired nor assumed in DFINE to have a ring-like manifold in data, as shown for example in the Torus or Swiss-roll simulations, neither of which has a ring-like manifold (Fig. 3). To conclude that the ring-like manifold is a good description of neural data, we needed to use the TDA metric combined with the neural prediction metric; together, these metrics show not only that there exists a ring-like manifold in DFINE latent trajectories but also that this manifold predicts the neural dynamics better in Figs. 4 and 5. Overall, these results show that the more predictive ring-like manifold structure was more robustly captured with DFINE.

### DFINE can also be extended to enable supervision and improve behaviour prediction accuracy

So far, we have presented the results of DFINE in which the model is trained unsupervised with respect to behaviour and to optimize neural prediction alone. To allow for considering continuous behaviour measurements when available during training, we next developed supervised DFINE to train a model with latent factors that are optimized for both neural and behaviour predictions simultaneously (Fig. 1a and Methods). To validate the supervised DFINE, we applied it to the motor datasets in which continuous behaviour measurements were available during training. Note that once trained, supervised DFINE inference only takes neural activity as input to infer the latent factors.

**Fig. 6:**
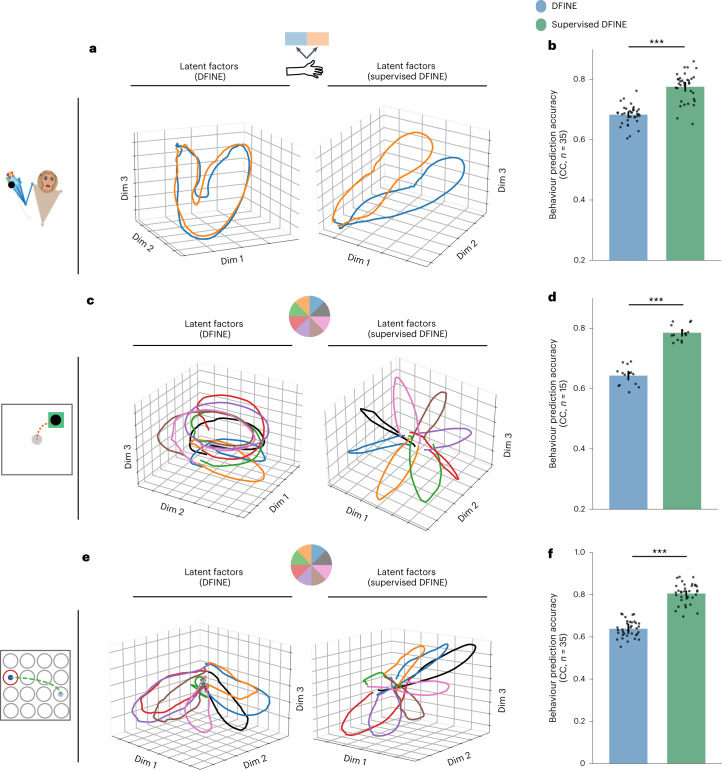
Supervised DFINE extracts latent factors that are more behaviour predictive. **a**, Examples of condition-average latent factor trajectories for DFINE (left) and its supervised version (right) are shown for the 3D reach-and-grasp task. Each colour represents one condition (that is, movement to the left or right; Methods). Supervised DFINE better separates different conditions. **b**, Supervised DFINE improves the prediction of behaviour, that is, continuous position and velocity, in the 3D reach-and-grasp task. Dots represent cross-validation folds across experimental sessions and the convention for bars, error bars and asterisks are the same as in Fig. 4. Similar results hold for the 2D random-target reaching task (**c**,**d**) and the 2D grid reaching task (**e**,**f**), where here each condition is a direction angle interval/sector (for example, all movements whose direction angle is between 0–45 degrees regardless of where they start/end; Methods). See Supplementary Fig. 5 for neural prediction accuracies.

We found that supervised DFINE improves the behaviour prediction accuracy even though its model and inference architectures are identical to those in DFINE, and its inference only takes the same neural observations as input (Fig. 6 and Methods). However, DFINE does better in terms of neural prediction accuracy (Supplementary Fig. 5). In particular, the latent factors of supervised DFINE better distinguished different task conditions across all motor datasets compared with those of DFINE (Fig. 6a,c,e). Consistent with this observation, supervised DFINE significantly improved behaviour prediction accuracy compared with DFINE across all motor datasets (Fig. 6b,d,f). These improvements were 13.6 ± 2.7% in the 3D naturalistic reach-and-grasp task (Fig. 6b; $P < 3.1\times {10}^{-5}$, *N*_s_ = 35, one-sided Wilcoxon signed-rank test), 22.3 ± 1.8% in the 2D random-target reaching task (Fig. 6d; $P < 2.6\times {10}^{-4}$, *N*_s_ = 15, one-sided Wilcoxon signed-rank test) and 24.5 ± 2.5% for the 2D grid reaching task (Fig. 6f; $P < 1.2\times {10}^{-7}$, *N*_s_ = 35, one-sided Wilcoxon signed-rank test). However, as expected, the neural prediction accuracy of supervised DFINE was significantly lower than that of DFINE across all datasets (Supplementary Fig. 5); this is because supervised DFINE’s latent factors are optimized for both behaviour and neural prediction simultaneously, while those of DFINE are optimized purely for neural prediction. Of note, the fact that behaviour variables are considered during model training does not definitively imply that latent factors extracted in the test set will be more behaviour predictive. This is because in the test set, the inference method for extraction is identical to that in DFINE but just with different parameter values, and the behaviour variables are still unknown during inference. Thus, this analysis shows that supervised DFINE learns a model with the same flexible inference properties that can this time extract more behaviour predictive latent factors from neural data.

### DFINE can perform flexible inference with missing observations

**Fig. 7:**
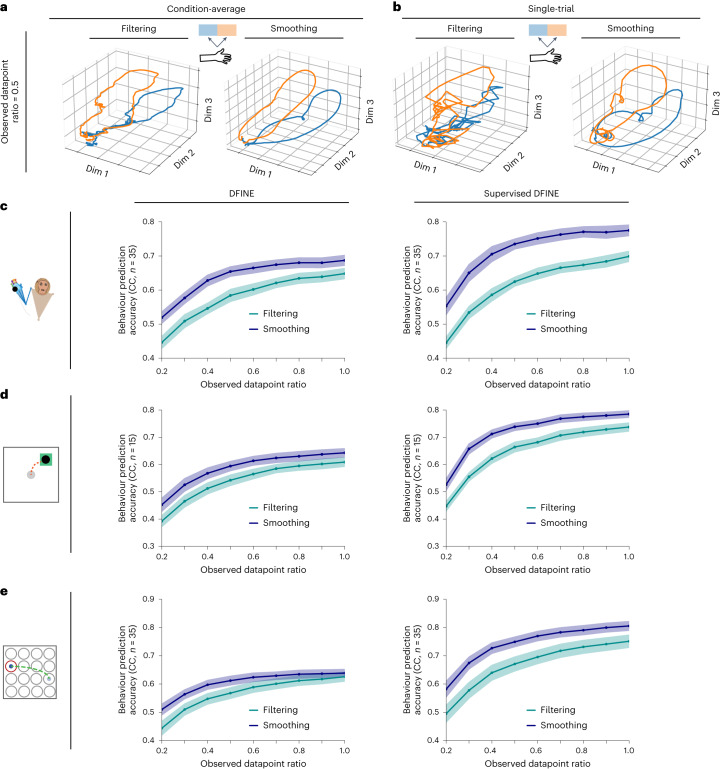
DFINE can perform both causal and non-causal inference with missing observations and do so more accurately through non-causal inference. **a**,**b**, Examples of condition-average (**a**) and single-trial (**b**) latent factor trajectories for filtering and smoothing inference with missing observations in the 3D reach-and-grasp task are shown. Both DFINE filtering and smoothing captures the low-dimensional structure in single trials despite the missing observations, with smoothing doing so more accurately than filtering. **c**, Behaviour prediction accuracies of filtering and smoothing inferences are shown across various observed datapoint ratios for the 3D naturalistic reach-and-grasp task. Lines represent the mean across experimental sessions and cross-validation folds, and the shaded areas represent the 95% confidence bound. **d**, Similar to **c**, for the 2D random-target reaching task. **e**, Similar to **c**, for the 2D grid reaching task. See also Extended Data Fig. 6, which shows that DFINE outperforms LDM and SAE when there are missing observations.

We next investigated whether DFINE can perform inference even in the presence of missing neural observations, which can for example commonly happen in wireless neural interfaces^24–31^ (Fig. 7). To do so, we uniformly dropped neural observations throughout the recordings (Methods) and performed inference in two ways: (1) filtering inference (causal) that uses only the past and present available neural observations and (2) smoothing inference (non-causal) that uses all available neural observations.

We found that behaviour prediction accuracies of DFINE remained relatively unchanged even when dropping up to 40% of observations (0.6 ratio) and remained well above the chance level of 0 even when dropping 80% of observations (lowest 0.2 ratio) (Fig. 7c–e; $P < 5\times {10}^{-4}$, one-sided Wilcoxon signed-rank test). Indeed, both filtering and smoothing inferences were robust to data drop. Also, behaviour prediction accuracy with smoothing inference was better than that with filtering inference across all observed datapoint ratios as expected (Fig. 7c–e). Figure 7a,b visually demonstrate that the smoothing inference yields more accurate reconstruction of the low-dimensional latent trajectories as it leverages both past and future available observations. Indeed, the smoothed trajectories at observed datapoint ratio of 0.5 (Fig. 7a) look very similar to those for observed datapoint ratio of 1 (Fig. 6a, right panel), showing ability to compensate for missing observations.

**Extended Data Fig. 6:**
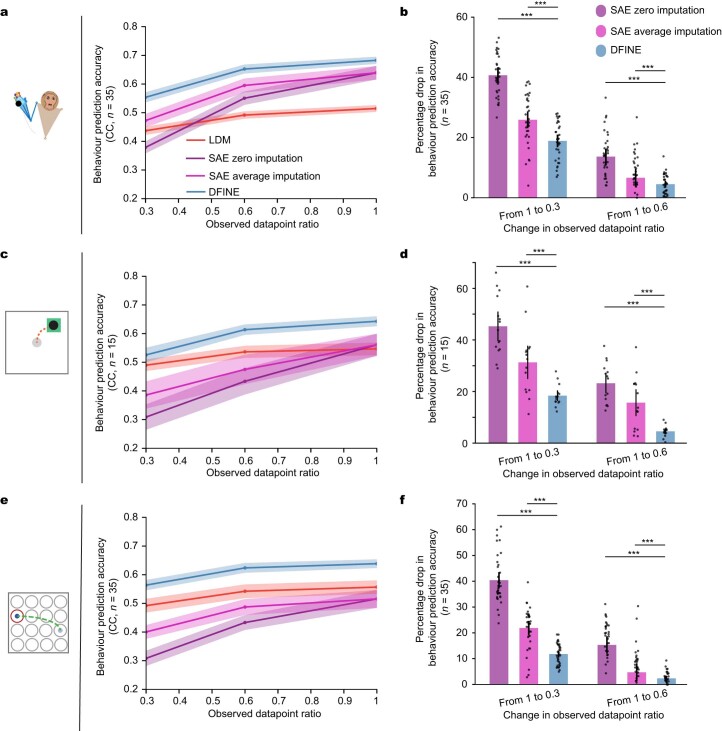
DFINE outperforms LDM and SAE in the presence of missing observations and the improvement vs. SAE grows with more missing samples. **a**, LDM, SAE and DFINE’s behaviour prediction accuracies across various observed datapoint ratios in the 3D naturalistic reach-and-grasp task. Figure convention is similar to that in Fig. 7. Given models trained on fully observed neural observations, these methods inferred the latent factors in the test set that had missing observations. LDM performed this inference with the Kalman filter/smoother. SAE did so either by imputing missing observations in the test set to zero as done previously^53,54^ (SAE zero imputation), or by imputing them to the average of the last and next/future seen observations (SAE average imputation). DFINE did so through its new flexible inference method. We then used the inferred factors in the test set to predict the behaviour variables. This process was done at 0.3 and 0.6 observed datapoint ratios. For all models, we show the behaviour prediction accuracy of the smoothed latent factors. DFINE’s behaviour prediction accuracy remains better than other models even in the lower observed datapoint ratios. **b**, The percentage drop in the behaviour prediction accuracy of the nonlinear models – SAE zero imputation, SAE average imputation and DFINE – as we vary the observed datapoint ratio from 1 to 0.3 and from 1 to 0.6. The percentage drop in behaviour prediction accuracy of DFINE is significantly lower than that of SAE with both imputation techniques, showing that DFINE can better compensate for missing observations. Figure convention for bars, dots and asterisks is similar to that in Fig. 4. Similar results held for the 2D random-target reaching task (**c, d**), and for the 2D grid reaching task (**e, f**).

We then compared DFINE with SAE in terms of inference in the presence of missing observations. Because SAE’s inference is performed with RNNs, it is structured to take neural observation inputs at every time step. To handle missing observations for SAE at inference, we imputed them with zeros in the test set as previously done^53,54^, extracted the latent factors and computed the associated behaviour prediction accuracy. DFINE outperformed SAE and, interestingly, this improvement grew larger at lower observed datapoint ratios (Extended Data Fig. 6). Indeed, DFINE’s degradation in performance due to missing observations was lower than that of SAE (Extended Data Fig. 6b,d,f). In a control analysis, we performed another imputation technique for SAE, where we imputed the missing observations with the average of the last and the next/future seen observation samples. Again, with this imputation technique, DFINE outperformed SAE (Extended Data Fig. 6) and showed more robustness as the observed datapoint ratio decreased (Extended Data Fig. 6b,d,f). In addition, DFINE’s performance remained above LDM’s performance even in the lower observed datapoint ratios (Extended Data Fig. 6a,c,e). These analyses show that DFINE can flexibly compensate for missing observations and perform both causal and non-causal inference with missing data. These analyses also show that DFINE’s non-causal inference can leverage future data for more accurate inference when real-time processing is not needed.

## Discussion

We developed DFINE, a neural-network model of neural population activity, and showed that it enables flexible inference, whether causally or recursively in real time, non-causally to leverage the entire data length, or in the presence of missing neural observations. DFINE jointly learned the combination of a nonlinear latent manifold and linear dynamics on top of this manifold, which together allowed it to achieve flexible inference and accurate prediction in diverse neural datasets and in nonlinear simulations including several manifolds and the Lorenz attractor system. This flexible inference capability is critical for the design of neurotechnology or wireless neural interfaces, and for the study of neural dynamics through causal closed-loop perturbations or through long-term or unconstrained experiments. In addition to enabling flexible inference, DFINE more accurately predicted both the neural and behavioural data, and more robustly discovered the low-dimensional neural manifold compared with linear and nonlinear benchmarks for smoothed firing rates and LFP in the four datasets. We also developed a supervised learning procedure for DFINE for cases where the goal is to extract latent factors that are more behaviour predictive, with no changes to the model architecture and inference properties otherwise. DFINE’s capabilities and advantages generalized across four independent datasets with different behavioural tasks, brain regions and neural signal modalities.

### DFINE allows for neural-network modelling while also enabling flexible inference

Many studies have shown that neural population activity can be summarized with a considerably lower-dimensional latent manifold structure^1,3–13,15,71–73^, often using linear dimensionality reduction methods^1,3,4,6,8–13,15,71,73^. To model the nonlinearities in neural population activity and better learn the underlying manifold structure^5–7,11,49–52,57,58,72,74–83^, while some studies have used spline loop fitting^7^ or extensions of isometric feature mapping (Isomap)^72^, most works have leveraged the power of neural networks^5,6,11,49–52,57,58,74–83^. As models of neural data, neural networks have been in the form of generative models^5,14,49–52,59,82^, whether static^82^ or dynamic^5,14,49–52,59^, or in the form of predictive models or decoders^57–59,81^. DFINE provides a dynamic generative model of neural data but with the major difference that it provides the new capability for flexible inference, in addition to being accurate in neural, behaviour and manifold prediction. DFINE has flexible inference in that it simultaneously enables recursive real-time/causal inference, non-causal inference and inference in the presence of missing observations. Such flexible inference is critical for neurotechnology design but is not achieved by previous neural-network models of population activity.

Previous generative network models do not provide these flexible inference properties. This is because their inference cannot be solved analytically and is usually performed with an inference/recognition network that is trained in conjunction with the generative network to approximate the posterior distribution of latent factors. Therefore, inference properties depend on how the inference network architecture is structured to process the neural observations. In many cases, the inference network structure does not allow for real-time recursive estimation of latent factors^5,14,49,50,52^, and/or does not directly enable inference in the presence of missing observations^5,14,50–52^. For example, SAEs^5^, which are often used as benchmarks for neural data, are most suitable for non-causal processing and are not necessarily amenable to recursive and/or causal/real-time inference in ongoing time-series as has been shown for example in natural language processing or speech recognition literature^84–86^. This is because of the SAE structure in which, with every new neural observation, the encoder RNN has to update the generator RNN’s initial latent state at time zero; then the generator RNN needs to reset and re-estimate its latent states throughout all time steps in a trial or data segment to infer the latent factor at the time of the new neural observation. This non-recursive procedure can be a large burden for real-time inference as the current estimate of latent factors cannot be utilized to get the next time-step’s factors. Indeed, all the latent factors have to be re-computed for every single new neural observation. In contrast, DFINE allows for recursive inference without the need to re-compute any of the past or current factors. Also, while the initial condition in autoencoders has a critical role by summarizing the time-series information and by enabling their powerful structure, the initial condition does not have an important role in DFINE or LDM and its effect disappears as the filter goes to steady state^87^. Finally, DFINE can directly perform training and inference on continuous data streams, which are encountered in non-trial-based or naturalistic datasets, in datasets with trials of different lengths, or in neurotechnologies such as BMIs. In terms of addressing missing observations, while imputation techniques such as zero-padding have been used for SAEs or in general for models with RNNs^53,54^, it is known that such techniques usually yield suboptimal performance^55,56^, as we also saw in our results (Extended Data Fig. 6). This is because imputing missing observations with zero in inference distorts the observation values. Similar to SAEs, in previous LDMs with nonlinear embeddings, that is, fLDS that we compared to^50^, inference again needs to use all neural observations and is non-causal; therefore, recursive causal inference or inference with missing observations is not directly addressed.

Similar to these previous generative networks, previous predictive networks with RNN-based methods^57–59^ also do not allow for flexible inference. This is because, this time, these networks do not enable non-causal inference given their forward RNN architecture and do not directly address missing neural observations as RNNs are structured to take inputs at every time step. Further, unlike DFINE, these predictive networks^57,58^ do not directly learn a generative model of neural population activity and thus are largely used for decoding purposes rather than to study/infer the latent neural population structure^57,58^.

Beyond biomedical engineering and neuroscience, dynamical modelling of sequential data has great importance for other domains such as for the processing of videos^88–90^ or text^91,92^, with great progress already made so far. However, neural data introduce distinct challenges that DFINE’s modelling architecture was specifically designed to handle. First, to consider the noisy nature of neural activity, we model and learn stochastic noise variables in DFINE. This allows us to model uncertainties and to fit the noise values to any specific dataset across diverse tasks, brain regions and neural data modalities (for example, firing rates or LFP). In contrast, because video or text observations are deterministically observed or are much less noisy, in applications of videos and text, the stochastic noise variables are not necessarily included in the modelling or are tuned manually as hyperparameters^88–92^. In addition, instead of the common training method of optimizing the evidence lower bound (ELBO) of data likelihood when noise variables exist^5,50,89^, we optimize the prediction accuracy of data into the future because optimizing ELBO could be challenging^84,93–95^ and a major goal of a dynamic model is future prediction; indeed, we observed instabilities when using ELBO as our optimization cost. We emphasize that the reason DFINE can leverage this future-step-ahead prediction as its training cost is its flexible inference capability that can efficiently and recursively compute this cost during training. Finally, unlike these video/text applications^88–92^, we also developed a supervised learning algorithm for DFINE to allow for the modelling of two different but related sets of sequential data (neural activity and behaviour here) and motivate the latent factors to be predictive of not just one but both time-series/sequences simultaneously. In addition to modelling of neural/behaviour data in neuroscience, this supervised learning algorithm could show promise for future applications of video/text data where another measured time-series/sequence is also of interest, for example, when inferring latent factors of videos regarding movements^96^.

### Linear temporal dynamics on the nonlinear manifold and extension across neural modalities

To extend the LDMs of neural population activity^1,3,12,13,21,22,44–47^, several previous studies have built nonlinear models of temporal dynamics using neural networks^5,49,51,52^ or Gaussian-process-based methods^97–100^. While DFINE develops a neural-network model with nonlinear manifold embeddings, it keeps the temporal dynamics on the manifold linear for several reasons. First, the separation of the dynamic and manifold latent factors and the linear form of the former is what allows for the flexible inference capability, which is critical for neurotechnology and various neuroscience applications. Second, since all manifold and dynamic model parameters are trained together in an end-to-end single optimization, DFINE learns the best nonlinear manifold over which dynamics can be approximated as linearly as possible. This combined manifold–dynamics approach allows DFINE to capture nonlinear temporal dynamics well, as we showed in simulations with the nonlinear stochastic Lorenz attractor system. Third, recent work that dissociates the source of nonlinearity in neural population activity has shown that linear dynamics in the presence of other nonlinearities may be sufficient in explaining the neural observations^59^. Our results suggest that the linear description of temporal dynamics on the nonlinear manifold in DFINE does not degrade the neural and behaviour prediction in these datasets, as DFINE outperforms fully nonlinear SAEs with nonlinear temporal dynamics (Figs. 4 and 5). This is despite the fact that these SAEs can have even more general nonlinear dynamics than the time-varying dynamics in LDM extensions, such as switching LDMs^101–103^. Finally, DFINE can be extended to allow for locally linear dynamics as described below to better capture potential nonlinearities in temporal dynamics that may not be fully captured using the joint optimization of the nonlinear manifold factors and linear dynamic factors.

In the DFINE model, we used a Gaussian distribution for neural observations such that our architecture can generalize across various commonly used neural data modalities including not only smoothed firing rates but also field potentials, which are continuous-valued. Indeed, field potentials such as LFP or electrocorticography are sometimes the only available modality in implantable neural interfaces and in many human neurophysiology experiments^27,40,41,43,104,105^. Also, field potentials can provide a robust modality for neurotechnologies as they can be long-lasting^17,106^ and can provide comparable information to spiking activity when decoding certain behaviour variables^12,13,68,107–109^. Field potentials can also reveal larger-scale network computations during behaviour for basic science investigations^12^. Beyond this Gaussian distribution, extending DFINE to support other observation distributions is a future direction.

A major objective in DFINE is to allow for dynamical flexible inference in its neural-network model, which is not achieved with previous nonlinear latent factor models of population activity including SAEs. This is critical because, regardless of performance, a nonlinear latent factor model requires flexible inference to be applicable in many neurotechnology applications and in many closed-loop or unconstrained neuroscience experiments. Nevertheless, to show that despite enabling this flexible inference capability DFINE still allows for accurate modelling and inference, we compared it with SAEs using the LFADS architecture^5^ as a benchmark (Methods). For a fair comparison, similar to DFINE, we had LFADS use a Gaussian observation distribution to make it applicable to both Gaussian-smoothed firing rates and field potentials (Gaussian observation distribution is enabled in the LFADS codebase and implementation). To get a conservative estimate of the improvements in DFINE, we allowed LFADS to have a higher dynamics state dimension than DFINE (64 vs 16; see Methods). For the choice of hyperparameters in LFADS, we picked the values given for one of the datasets in the original work^5^ that had the closest number of trials to our datasets (Methods). It is possible that a comprehensive hyperparameter tuning can improve LFADS’s performance; however, this tuning would require prohibitive training time/resource, given the complex architecture and its large number of hyperparameters. While an extension of LFADS, AutoLFADS^110^, could help with this tuning, the existing AutoLFADS implementation during our developments does not support Gaussian observation distributions unlike LFADS and DFINE, and thus is not directly comparable to DFINE or applicable to the field potential modalities here. Furthermore, AutoLFADS optimizes only the non-architectural hyperparameters during training, and the architectural hyperparameter search (such as all layer/RNN latent state dimensions) still requires considerable training resource/time. In contrast, DFINE uses the same architectural hyperparameters for all datasets in both its unsupervised (default) and supervised versions, thus not requiring such an extensive search. This shows the simple and generalizable architecture and training of DFINE. Moreover, AutoLFADS is again an SAE, which poses a challenge for flexible inference and does not perform it as discussed above. Nevertheless, to test the choice of LFADS hyperparameters here, we applied LFADS with these hyperparameters to a publicly available spiking dataset during a maze task^111^ that has recently been tested with AutoLFADS. We found that LFADS with the chosen hyperparameters here had a behaviour decoding performance similar to that reported for AutoLFADS on this same maze data^111^, suggesting the appropriateness of the chosen LFADS hyperparameters here.

The Lorenz simulation in Extended Data Fig. 4 suggests that DFINE outperforms SAE/LFADS on our neural datasets potentially because of inherent stochasticity in neural dynamics and the inevitable model mismatches that also introduce uncertainty, which can be captured via stochastic noise variables (no model is perfect for data). Indeed, DFINE performed better than SAE when there was some stochasticity in the Lorenz dynamics, while SAE could do better when dynamics were almost deterministic. We emphasize however that, regardless of performance, DFINE and SAE/LFADS have distinct yet complementary goals and so will have utility in complementary applications. DFINE’s focus is on biomedical engineering applications such as BMIs and implantable/wireless neural interfaces; further, DFINE focuses on neuroscience studies that involve causal closed-loop validation/perturbation or require longer-term recordings or unconstrained setups. LFADS is a powerful SAE architecture that has utility for basic neuroscience investigations (for example, for trial-based spike datasets).

DFINE also outperformed LDM because of incorporation of embedding nonlinearities. Further, DFINE outperformed another benchmark method termed fLDS that has linear dynamics and a direct nonlinear observation model^50^ (Extended Data Fig. 3). DFINE is different from fLDS in terms of model architecture, inference procedure and learning cost function. DFINE’s model architecture is different from that in fLDS because DFINE has a two-layer factor architecture with two distinct factors rather than the one-layer factor in fLDS, and because DFINE incorporates stochastic nonlinearity on its dynamic factors while fLDS has deterministic nonlinearity (Methods). This architecture change is why DFINE can enable flexible inference unlike fLDS, can optimize the distinct future-step-ahead prediction as the learning cost function while fLDS uses the ELBO cost, and can ultimately improve performance on neural data compared with fLDS (Extended Data Fig. 3). Indeed, DFINE can swiftly compute the future-step-ahead prediction error cost during learning because of its new capability for flexible recursive inference, without which using this cost is not practically feasible (Supplementary Table 1). As such, the reasons why DFINE enables flexible inference are the above architectural differences including the additional noisy manifold layer. These differences combined with the distinct learning cost in DFINE also lead to the performance improvements of DFINE over fLDS, potentially in part due to the known issues with the trade-off between the reconstruction term and the Kullback–Leibler penalty term in the ELBO cost used by fLDS^84,93–95^.

It is also important to note that the two-layer factor design in DFINE is distinct from applying data preprocessing, for example, in the form of principal component analysis, before model learning to mitigate model overfitting^43^. In particular, the manifold embedding encoder/decoder in DFINE is nonlinear and further is learned end-to-end in conjunction with the linear dynamical part of the model (as distinct from separate preprocessing whether linear or nonlinear). This joint manifold–dynamics learning enforces the encoder network to learn the best nonlinear manifold embedding over which dynamics can be described well with an LDM. The joint learning also helps with the difficult problem of identifying a nonlinear manifold in noisy data through dynamic denoising over time. We showed that this joint manifold–dynamics learning allows DFINE to capture the latent trajectories of a system with nonlinear temporal dynamics in the Lorenz simulations while still allowing for flexible inference (Extended Data Fig. 4).

### Revealing low-dimensional manifold structure in the saccade and motor datasets

Latent factor models have led to important insight about neural population coding during various behavioural tasks. For example, rotatory low-dimensional structure in the neural population activity has been prevalently observed^1,3,5–7,9–13,15,69,76,112,113^, in the motor system during movements^1,3,5,10–13,69,76,112,113^ as well as other systems during tasks such as a syllables task^9^, in a ready-set-go task while performing a saccade^6^, in an exploration task in the head direction system^7^, and during auditory stimulation^15^. Here, DFINE found latent factors that consistently had ring-like manifold structures as revealed by visualization and TDA analysis and that were more predictive of neural activity and behaviour, thus suggesting they were good descriptions of neural data. Importantly, DFINE extracted such ring-like manifold structures more robustly in single trials compared with LDM and SAE in all datasets. Moreover, compared with alternative models, DFINE required fewer latent factors to predict neural population activity with a given accuracy (Supplementary Fig. 1), showing that DFINE is also useful for unsupervised dimensionality reduction in neural datasets.

In addition to allowing for flexible inference, the separation of dynamic and manifold factors in DFINE can facilitate neuroscientific interpretation. We can interpret the manifold latent factors as capturing the global latent structure of the population code and the dynamic latent factors as revealing its local properties and variations. This is because the same manifold can be traversed in many ways, which are captured by the dynamic latent factors; thus, dynamic factors may correspond to local changes in behaviour, while manifold factors may relate to global changes in behaviour. Combined with its accurate and robust discovery of the latent structure, this separation can also facilitate the use of DFINE for investigations and interpretations across diverse domains of neuroscience.

### The applications of flexible inference enabled in DFINE

A major contribution of DFINE compared with previous nonlinear latent factor models is that it enables the capability for flexible inference. Regardless of performance, flexible inference is crucial to enable the use of nonlinear latent factor models in many critical biomedical engineering applications such as real-time neurotechnology, BMIs or advanced implantable/wireless neural interfaces^17,24,27,30,35–42^. Flexible inference is also important for neuroscience studies that involve causal or closed-loop perturbations/validations, long-term monitoring of brain states (for example, mood^43^) or behaviours in unconstrained environments^17,22,24,27,30,32–43^, as further expanded on below. In addition, flexible inference is critical in model-based closed-loop control of neurostimulation, for example, in mental disorders^17,22,33^. Further, we show that DFINE performs accurately not only for neuronal firing rates but also for field potentials, which are a critical modality for biomedical engineering and human neurophysiology^12^^,22,27^^,40^^,41,43,104,105^.

One major feature of DFINE is enabling inference in the presence of missing neural observations, which is a major challenge in wireless implantable neural interfaces due to intermittent wireless disruptions that cause all neural observations from all channels to be lost at random disruption times. These random disruptions have been shown to occur in recent wireless BMIs or DBS systems, for example, due to the appearance of other individuals, signals around the user’s environment, or in general any random event breaking the wireless transmission^24–31^. We emulated this random disruption scenario in our data drop analyses by making the probability of data loss approximately equal for every timepoint. We found that DFINE makes the inference robust to such data loss. Thus, this capability of DFINE is important to facilitate the use of powerful nonlinear decoders for wireless BMIs or closed-loop DBS technologies that can be utilized in daily life. Further, in neuroscience, there is recent interest to study more naturalistic behaviours in unconstrained environments or to investigate brain states that have slow timescales such as mood^43^ or chronic pain. In both cases, having the user tethered to the decoding system is infeasible or difficult, and transition to wireless interfaces is imperative to enable operation in unconstrained environments and/or over long timescales (for example, for 24/7 monitoring or therapy), improve user mobility and convenience during daily/naturalistic tasks, and minimize risks of infection^24,27,30,35–42^.

DFINE allows the same model to perform both real-time causal and non-causal inference. This capability is important in moving from correlational validations of a model of neural population dynamics to its causal validation^32^. Given the challenges of causal real-time studies, many neuroscientific studies rely on correlative analysis to build knowledge about the associations between a given neural model and behavioural variables. However, the next-level validation of the same model will be to establish the causal associations between its latent factors and behavioural variables, for example, through causal closed-loop perturbation experiments^17,22,33^. Since the goal is to validate the same model, this same model should be used to infer the latent factors in real time and use them as feedback to decide on the perturbation, modulate the latent factors and see the causal effect of such closed-loop modulation on behaviour^17,22,32–34^. Thus, ideally, we would like models that not only enable offline and accurate non-causal inference of brain states to test various correlative hypotheses, but also allow causal investigations and closed-loop perturbations enabled by real-time flexible inference. In addition, training generative models with deep learning requires considerable resources and hyperparameter tuning, which becomes a challenge especially for chronic neurotechnologies in which models need to be retrained often to track adaptation and plasticity, for example, due to electrical stimulation^114,115^. Instead of training different models, DFINE can just train the same model for both causal and non-causal inference. Finally, flexible inference is important to enable model-based closed-loop control with electrical stimulation in brain disorders^17,22^. This is because flexible inference allows offline neural biomarker models of symptom states to be used in a real-time closed-loop control paradigm to take the neural biomarker to a target level, which is a critical goal^17,22,33,34^.

## Future directions

DFINE enables flexible inference in its neural-network model. However, there are limitations that can be directions for future extensions. To enable flexible inference, DFINE models the nonlinear dynamics in the space of neural population activity through the combination and joint training of a nonlinear manifold embedding and linear dynamics on top of this embedded manifold. In addition to the four neural datasets here, we showed that this manifold–dynamics combination can predict the data in the Lorenz simulation even though the Lorenz system’s temporal dynamics are nonlinear and are not generated from the DFINE model, that is, exhibit model mismatch (Extended Data Fig. 4). However, it is possible that DFINE learns suboptimal results in some datasets due to its linearity of dynamics on the nonlinear manifold, which is a design choice made to enable flexible inference. Future work can extend DFINE to learn piece-wise linear dynamics on the manifold to improve the modelling, which still keeps the flexible inference capability intact. Further, an important capability for neuroscience studies is to combine data across multiple experiments/sessions, which is currently not implemented for DFINE and could be added/tested in future work. Finally, we developed DFINE with a Gaussian distribution for output neural observations to be generalizable across various neural modalities, whether smoothed firing rates or field potentials, which is critical in biomedical engineering applications. Extending DFINE to other output distributions, such as generalized linear models including Poisson/point process likelihoods^12,45–47,50,110,116^ or multiscale likelihoods^12,103,117–120^, is an important future direction. Finally, inputs could also be modelled in the future by incorporating them in the LDM part of the model, for example, for stimulation applications^17,22,121^.

## Outlook

Taken together, DFINE provides a neural network that enables the new capability for flexible inference of low-dimensional nonlinear latent factors in neural population activity. Further, DFINE achieves accurate neural, behaviour and manifold prediction. As such, DFINE can be used for developing enhanced neurotechnology in biomedical engineering and for causally probing neural population activity in neuroscience.

## Methods

### Neural data recordings

**Extended Data Fig. 7:**
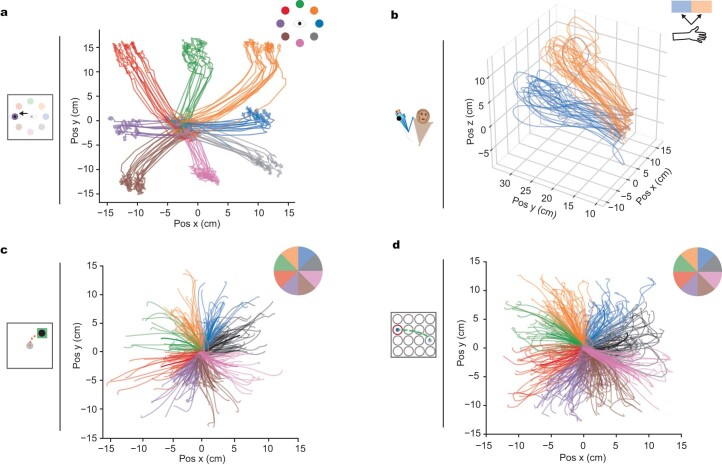
Example behaviour trajectories for the four experimental datasets. **a**, Eye movement trajectories for the saccade task. Each colour represents one target, that is, condition. **b**, 3D hand movement trajectories for the 3D naturalistic reach-and grasp task. Each colour represents one condition, that is, movement to the left or right. **c**,**d**, 2D cursor trajectories for the 2D random-target reaching task (**c**) and 2D grid reaching task (**d**) are shown, when shifted in space to start from the center. Each condition is shown with a different colour and represents reaches that have similar direction angles. Regardless of start or end position, the angle of movement specifies the 8 conditions, which correspond to movement angle intervals of 0–45, 45–90, 90–135, 135–180, 180–225, 225–270, 270–315, and 315–360, respectively.

We performed our analyses on four diverse datasets containing distinct behavioural tasks, brain regions and neural signal modalities to show the generalizability of the results (Fig. 2 and Extended Data Fig. 7).

#### Saccade task

A macaque monkey (Monkey A) performed saccadic eye movements during visually guided oculomotor delayed response task^67^ (Fig. 2a and Dataset 1). Each experimental session (13 sessions in total) consisted of several trials towards one of eight peripheral targets on a screen. All surgical and experimental procedures were performed in compliance with the National Institutes of Health (NIH) Guide for Care and Use of Laboratory Animals and were approved by the New York University Institutional Animal Care and Use Committee (IACUC). Trials began with the illumination of a central fixation square. The subject was trained to maintain its eyes on the square for ~500–800 ms. After this baseline period, a visual cue was flashed for 300 ms at one of the eight peripheral locations to indicate the target of the saccade (Target On event). After a delay, the central fixation square was extinguished, indicating the Go command to start the saccade (Go event). The subject was trained to perform the saccade (Saccade Start event) and maintain fixation on the target for an additional 300 ms. A fluid reward was then delivered. The visual stimuli were controlled via custom LabVIEW (National Instruments) software. Eye position was tracked with an infrared optical eye tracking system (ISCAN) and from these positions, some of the task events such as Saccade Start were identified. In this task, there were eight task conditions, each representing trials to one of the eight targets. During the task, LFP signals were recorded from lateral PFC with a semi-chronic 32-microelectrode array microdrive (SC32-1, Gray Matter Research). Raw LFP signals were low-pass filtered at 300 Hz and downsampled to 20 Hz leading to an LFP observation every 50 ms.

#### Naturalistic 3D reach-and-grasp task

A macaque monkey (Monkey J) performed a naturalistic reach-and-grasp task in a 50 × 50 × 50 cm^3^ workspace for a liquid reward across seven experimental sessions (Fig. 2b and Dataset 2). All surgical and experimental procedures were performed in compliance with the NIH Guide for Care and Use of Laboratory Animals and were approved by the New York University IACUC. During the task, the subject naturalistically reached for an object positioned on a wand, grasped it, released it and then returned its hand to a natural resting position^12^. The wand was continuously moved around by the experimenter within a diverse spatial area in front of the subject^12^. The task was performed continuously in time without any instructions to isolate reach-and-grasp movement components. A total of 23 retroreflective markers were attached on the subject’s right arm and monitored using infrared and near-infrared motion capture cameras (Osprey Digital RealTime System, Motion Analysis Corp.) at a sampling rate of 100 frames per second. We labelled 3D marker trajectories on the arm and hand (Cortex, Motion Analysis Corp.). The behaviour variables were taken as the arm kinematics, that is, the position and velocity of the wrist marker in the *x*, *y* and *z* directions. On each frame, motion capture camera data acquisition was synchronized to the neural recordings using a synchronization trigger pulse. The task lacked pre-defined trial structure or pre-defined target locations. Therefore, for visualization purposes and after performing modelling and inference on continuous data, we identified the trial starts and ends from the velocity of the hand movement^12^, where the start of the trials was set to the start of the reach, and the end of the trials was set as the end of return and hold durations of the movement (Fig. 2b). To show condition-average visualizations, we partitioned the trials into two different conditions corresponding to leftward or rightward reaches along the horizontal axis in front of the subject, respectively. The horizontal axis was chosen for this division because it explained the largest variability in the reach locations.

Neural activity was recorded from M1, PMd, PMv and PFC in the left (contralateral) hemisphere with an array containing 137 microelectrodes (large-scale microdrive system, Gray Matter Research). Similar to our previous work^12^, we analysed the pool of top 30 spiking channels sorted on the basis of the individual channel behaviour prediction accuracies. Gaussian-smoothed firing rates were calculated by counting the spikes in 10 ms bins and applying a causal Gaussian kernel smoother^71,73,97,122,123^ (with 30 ms standard deviation), followed by downsampling to have observations every 50 ms. We also analysed raw LFP signals from the pool of top 30 LFP channels sorted on the basis of the individual channel behaviour prediction accuracies. Raw LFP signals were low-pass filtered at 400 Hz and downsampled to 20 Hz leading to an LFP observation every 50 ms.

#### 2D random-target reaching task

A macaque monkey (Monkey T) performed a 2D random-target reaching task with an on-screen cursor in a total of three experimental sessions, data for which are publicly available^63,64^ (Fig. 2c and Dataset 3). All surgical and experimental procedures were consistent with the guide for the care and use of laboratory animals and approved by the IACUC of Northwestern University^63,64^. The subject controlled the cursor using a two-link planar manipulandum while seated in a primate chair. Hand movements were constrained to a horizontal plane within a 20 × 20 cm^2^ workspace. The task consisted of several sections in each of which the subject performed 4 sequential reaches to random visual targets that appeared on the screen to receive a liquid reward (Fig. 2c). Within each section, after reaching the target and holding for a short period, the next target appeared in a pseudo-random location within a circular region (radius = 5–15 cm, angle = 360 degrees) centred on the current target. On average, the next target appeared ~200 ms after the subject reached the current target. The task naturally consisted of non-stereotyped reaches to different locations on a 2D screen unlike traditional centre-out cursor control tasks with stereotyped conditions^63^. Here, 2D cursor position and velocity in *x* and *y* directions were used as behaviour variables. To show condition-average visualizations, we partitioned the reaches into 8 different conditions, given the direction angle between the start and end point of the cursor trajectory (Extended Data Fig. 7). The angle of movement specifies the 8 conditions, which correspond to movement angle intervals of 0–45, 45–90, 90–135, 135–180, 180–225, 225–270, 270–315 and 315–360, respectively.

The subject was implanted with a 100-electrode array (Blackrock Microsystems) in PMd. After spike sorting, two sets of units were excluded from analysis in the original work^63^: (1) the units with low firing rates (smaller than 2 spikes per second) and (2) the units that had high correlations with other units. This led to 46, 49 and 57 number of units across different recording sessions. Gaussian-smoothed firing rates were calculated by counting the spikes in 10 ms bins and applying a causal Gaussian kernel smoother (with 30 ms standard deviation), followed by downsampling to have observations every 50 ms.

#### 2D grid reaching task

A macaque monkey (Monkey I) performed a 2D grid reaching task by controlling a cursor on a 2D surface in a virtual-reality environment, data for which are publicly available^65,66^ (Fig. 2d and Dataset 4). All animal procedures were performed in accordance with the US National Research Council’s Guide for the Care and Use of Laboratory Animals and were approved by the UCSF IACUC^65,66^. Circular targets with 5 mm visual radius within an 8 × 8 square grid or an 8 × 17 rectangular grid were presented to the subject and the cursor was controlled with the subject’s fingertip position. Fingertip position was monitored by a 6-axis electromagnetic position sensor (Polhemus Liberty) at 250 Hz and then non-causally low-pass filtered to reject sensor noise (4th order Butterworth filter with 10 Hz cut-off). The subject was trained to acquire the target by holding the cursor in the respective target-acceptance zone, a square of 7.5 mm edge length centred around each target, for 450 ms. After acquiring the target, a new target was drawn from the possible set of targets. In most sessions, this set was generated by replacement, that is, the last acquired target could be drawn as the new target. However, the last acquired target was removed from the set in some sessions. Even though there was no inter-trial interval between consecutive reaches, there existed a 200 ms ‘lockout interval’ after target acquisition where no new target could be acquired. 2D cursor position and velocity in *x* and *y* directions were used as behaviour variables. To show condition-average visualizations after modelling and inference on continuous data, we partitioned the reaches into 8 different conditions on the basis of their direction angle as in the 2D random-target reaching task.

One 96-channel silicon microelectrode array (Blackrock Microsystems) was implanted into the subject’s right hemisphere (contralateral) M1. A total of seven sessions (sessions 20160622/01 to 20160921/01) were used here^59^. We analysed the pool of top 30 neurons sorted on the basis of the individual neuron behaviour prediction accuracies. Gaussian-smoothed firing rates were calculated by counting the spikes in 10 ms bins and applying a causal Gaussian kernel smoother (with 30 ms standard deviation), followed by downsampling to have observations every 50 ms.

### DFINE model

We developed a neural-network architecture that allows for nonlinear description of neural data similar to deep neural networks, but in a manner that enables flexible inference (similar to LDM). This was done by combining a nonlinear latent manifold structure with linear dynamics on it and training them together to optimize a future-step-ahead prediction cost. In particular, we introduced two distinct sets of latent factors in the neural network for *n*_*y*_-dimensional neural population activity ${\bf{y}}_{t}\in {\mathbb{R}}^{{n}_{y}\times 1}$: dynamic latent factors ${{\bf{x}}}_{t}\in {{\mathbb{R}}}^{{n}_{{x}}\times 1}$ and manifold latent factors ${{\bf{a}}}_{t}\in {{\mathbb{R}}}^{{n}_{{a}}\times 1}$, where *n*_*x*_ and *n*_*a*_ are the factor dimensions/hyperparameters to be picked. A key idea is to incorporate a middle noisy manifold layer **a** between the dynamic latent factors **x** and neural observations **y**, which allows us to separate the model into a nonlinear manifold component and a linear dynamical component evolving on this nonlinear manifold (Fig. 1a). This separation plays a key role by enabling the new flexible inference capability of the network to optimally perform: (1) recursive causal inference (filtering), (2) non-causal inference with all neural observations (smoothing) and (3) inference in the presence of missing neural observations. Specifically, the separation enables flexible inference using a Kalman filter for the bottom dynamic part of the model in Fig. 1a that infers the dynamic factors **x** from the nonlinear manifold factors **a** (Fig. 1b). Another key idea is to optimize a future-step-ahead prediction cost during learning, which is enabled by flexible inference (see ‘The learning problem’ section below). We now describe the network architecture consisting of the dynamic and manifold factors.

First, the dynamic latent factor evolves in time with a linear Gaussian model:

1 $${{\bf{x}}}_{t+1}={A}{{\bf{x}}}_{t}+{{\bf{w}}}_{t},$$

where ${{\bf{w}}}_{t}\in {{\mathbb{R}}}^{{n}_{{x}}\times 1}$ is a zero-mean Gaussian noise with covariance matrix ${W}\in {{\mathbb{R}}}^{{n}_{{x}}\times {n}_{{x}}}$, and ${A}\in {{\mathbb{R}}}^{{n}_{{x}}\times {n}_{{x}}}$ is the state transition matrix. The manifold latent factor **a**_*t*_ is related to the dynamic latent factor **x**_*t*_ as:

2 $${{\bf{a}}}_{t}={C}{{\bf{x}}}_{t}+{{\bf{r}}}_{t},$$

where ${C}\in {{\mathbb{R}}}^{{n}_{{a}}\times {n}_{{x}}}$ is the emission matrix and ${{\bf{r}}}_{t}\in {{\mathbb{R}}}^{{n}_{{a}}\times 1}$ is a white Gaussian noise with covariance matrix ${R}\in {{\mathbb{R}}}^{{n}_{{a}}\times {n}_{{a}}}$. Equations (1) and (2) together form an LDM, with **a**_*t*_ being the Gaussian noisy observations. We denote the parameter set of this LDM by $\psi =\{{A},\,{W},\,{C},\,{R},\,{{\mathbf{\upmu}}}_{0},\,{{\varLambda}}_{0}\}$, where **μ**_0_ and *Λ*_0_ are the initial estimate and covariance of the dynamic latent factors, respectively.

Second, to model nonlinear mappings, we used autoencoders to learn the mapping between neural observations **y**_*t*_ and manifold latent factors **a**_*t*_. In general, autoencoders are static generative models made of two parts: the encoder that maps the observations to a bottleneck representation and the decoder that takes this bottleneck representation to the observations. Here, autoencoder observations are the neural observations and autoencoder bottleneck representation is given by the manifold latent factors. We model the decoder part as a nonlinear mapping *f*_*θ*_(⋅) from manifold latent factors to neural observations:

3 $${{\bf{y}}}_{t}={{f}}_{\theta }\left({{\bf{a}}}_{t}\right)+{{\bf{v}}}_{t},$$

where *θ* are parameters and ${{\bf{v}}}_{t}\in {{\mathbb{R}}}^{{n}_{{y}}\times 1}$ is a white Gaussian noise with covariance ${V}\in {{\mathbb{R}}}^{{n}_{{y}}\times {n}_{{y}}}$. We modelled nonlinear mappings with MLPs as they are universal approximators of any nonlinear function under mild conditions^60^. Equations (1–3) together form the generative model (Fig. 1a). For inference (next section), we also need the mapping from **y**_*t*_ to **a**_*t*_, which we characterize as:

4 $${{\bf{a}}}_{t}={{f}}_{\phi }({{\bf{y}}}_{t})$$

where *f*_*ϕ*_(⋅) represents the encoder in the autoencoder structure and is parameterized by another MLP (Fig. 1a).

It is important to emphasize that equations (1–4) are trained together and end-to-end (rather than separately). Further, we note that the middle manifold layer in equation (2) explicitly incorporates a stochastic noise variable **r**_*t*_, whose covariance is learned during training. This stochastic noise variable allows for the nonlinearity with respect to the dynamic latent factors to be stochastic in DFINE, that is, nonlinear function *f*_*θ*_(⋅) is stochastic with respect to **x**_*t*_ as it also has noise **r**_*t*_ inside it. Finally, to help with robustness to noise and stochasticity during inference, DFINE learns stochastic noise variables during training, which are then explicitly accounted for at inference as discussed in ‘The inference problem’ section below (see also stochastic Lorenz simulations in Extended Data Fig. 4).

### The inference problem

Using the model in equations (1–4), we needed to infer both the manifold and dynamic latent factors from neural observations **y**_1:*T*_, where *T* is the total number of time steps for the observations. We used the subscript *t*|*k* to denote the inferred latent factors at time *t*, given **y**_1:*k*_. Therefore, *t*|*t* denotes filtering (causal) inference given **y**_1:*t*_ and *t*|*T* denotes smoothing (non-causal) inference given **y**_1:*T*_. As an intermediate step called nonlinear manifold embedding, we first directly but statically obtained an initial estimate of **a**_*t*_ based on **y**_*t*_ from equation (4) as ${\hat{{\bf{a}}}}_{t}={f}_{\phi }({{\bf{y}}}_{t})$ to provide the noisy observations of the dynamical model, that is, ${\hat{{\bf{a}}}}_{t}$, in Fig. 1b. Having obtained ${\hat{{\bf{a}}}}_{t}$, we can now use the dynamical part of the model in equations (1) and (2) to infer **x**_*t*|*t*_ with Kalman filtering from ${\hat{{\bf{a}}}}_{1:t}$, and infer **x**_*t*|*T*_ with Kalman smoothing^124^ from ${\hat{{\bf{a}}}}_{1:T}$. We can then infer the manifold latent factor as ${{{\bf{a}}}_{t{{|}}t}={C\bf{x}}}_{t{{|}}t}$ and **a**_*t*|*T*_ **=** *C***x**_*t*|*T*_ on the basis of equation (2).

Note that **a**_*t*|*t*_ is inferred not only on the basis of the neural observations but also on the basis of the learned dynamical model using the Kalman filter, and thus this inference aggregates information over time dynamically. Given this procedure, DFINE’s inference explicitly accounts for the stochastic noise variables during inference by using them to optimally combine the dynamical model and the noisy neural observations in inferring the latent factors. This helps DFINE perform robustly and well in the presence of stochasticity in neural dynamics in the stochastic Lorenz simulations in Extended Data Fig. 4. Further, in general, identifying a nonlinear manifold embedding in noisy data is challenging. DFINE aims to facilitate this identification by learning the manifold embedding (MLP) and dynamical model jointly together, which also allows it to then infer the manifold latent factors dynamically in time and denoise them over time (that is, via **a**_*t*|*T*_ = *C***x**_*t*|*T*_).

The inference above also has the following major advantages compared with previous generative neural-network models of neural activity that train a non-causal inference network and use all observations in a trial/segment^5,50^. First, we can infer the latent factors recursively in real time, that is, use the current inferred dynamic latent factors **x**_*t*|*t*_ to calculate the next time-step’s inferred factors **x**_*t*+1|*t*+1_; this recursive nature also considerably reduces computation time (Supplementary Table 1). Second, we can handle missing observations by performing only forward prediction with the Kalman predictor^12,48,125^ at time steps when observations are missing, without any need to impute 0 values for these missing observations as done previously^53,54^. Third, we can perform both causal filtering and non-causal smoothing inference with the same learned model. Fourth, for datasets where there is no trial structure or trials are of different lengths, we can directly perform inference (and training) on continuous data time-series unlike inference in SAEs that requires having trials and/or segmenting the data to mimic trial-structured data (see ‘Implementation details for benchmark methods’).

### The learning problem

Neural-network model parameters are learned to optimize a cost function. When stochastic noise variables exist^5,50,89^, this cost is typically taken as the ELBO. But optimizing ELBO can be difficult^84,93–95^ and the direct goal of dynamical modelling is to predict neural and/or behaviour dynamics. Thus, we instead defined our cost as the *k*-step-ahead prediction error in predicting neural observations *k* time-steps into the future, that is, the error between ${{\bf{y}}}_{t{+}k}$ and its prediction from ${{\bf{y}}}_{1:t}$ denoted by ${{\bf{y}}}_{t{+}{k|t}}$. What allows us to utilize this cost is that our model enables efficient recursive inference/prediction to compute the *k*-step-ahead prediction error during training in the presence of noise (Fig. 1b); this is because we can run all forward predictions with a single run of Kalman filtering. Thus, our cost *L* is a function of all parameters as:

5 $$L(\psi ,\theta ,\phi )=\mathop{\sum }\limits_{k=1}^{K}\mathop{\sum }\limits_{t=1}^{T-k}e({{\bf{y}}}_{t+k{{|}}t},{{\bf{y}}}_{t+k})+{\lambda }_{{\rm{reg}}}{L}_{2}\left(\theta ,\phi \right),$$

where *K* denotes the maximum horizon for future-step-ahead prediction and *e*(·,·) denotes the error measure. *T* is the length of the time-series, that is, the length of each batch in the mini-batch gradient descent^126^. *ψ* is the set of LDM parameters in equations (1) and (2), that is, $\psi =\{{A},\,{W},\,{C},\,{R},\,{{\mathbf{\upmu}}}_{0},\,{{\varLambda}}_{0}\}$. *L*_2_(*θ*, *ϕ*) is an *L*_2_ penalty for the autoencoder parameters {*θ*, *ϕ*} to prevent overfitting with regularization hyperparameter *λ*_reg_^126^. In practice, we use MSE for the error measure *e*(·,·).

### Supervised DFINE learning

In DFINE, latent factors were optimized to be predictive of neural population activity, that is, learning is unsupervised by default. We also developed a supervised learning algorithm for DFINE that aims to extract latent factors that are more predictive of behaviour. We call the resulting algorithm supervised DFINE. In particular, when continuous behaviour variables ${{\bf{z}}}_{t}\in {{\mathbb{R}}}^{{n}_{{z}}\times 1}$ were available during training, we used them to supervise the training and learn latent factors that are predictive of both neural observations and behaviour variables simultaneously. This was done by adding an auxiliary neural network, termed mapper MLP network, to the DFINE graphical model during training only (Fig. 1a); this mapper network mapped the manifold latent factor **a**_*t*_ to behaviour variables **z**_*t*_ during training (the link from **a** to **z** in Fig. 1a) and is written as **z**_*t*_ = *f*_*γ*_(**a**_*t*_)+**q**_*t*_, where ${{\bf{q}}}_{t}\in {{\mathbb{R}}}^{{n}_{{z}}\times 1}$ is white Gaussian noise. To motivate the network to learn latent factors that are predictive of both behaviour and neural observations simultaneously, we added the behaviour prediction error to the single cost function in equation (5) as follows:

6 $$L(\psi ,\theta ,\phi ,\gamma )=\mathop{\sum }\limits_{k=1}^{K}\mathop{\sum }\limits_{t=1}^{T-k}e({{\bf{y}}}_{t+k{{|}}t},{{\bf{y}}}_{t+k})+{\lambda }_{{\rm{beh}}}\mathop{\sum }\limits_{t=1}^{T}e({f}_{\gamma }({{\bf{a}}}_{t}),{{\bf{z}}}_{t})+{\lambda }_{{\rm{reg}}}{L}_{2}\left(\theta ,\phi ,\gamma \right),$$

where *λ*_beh_ is a hyperparameter. Larger values of *λ*_beh_ put more emphasis on behaviour prediction vs neural prediction and vice versa. Note that the parameters of the auxiliary MLP (*γ*) were also added to the regularization term in the cost function.

We emphasize that after training of supervised DFINE was completed, the mapper MLP was discarded and not used for inference. The inference of latent factors remained identical to that in DFINE, and was done purely on the basis of neural observations and was independent of behaviour variables (Fig. 1b). The only difference was that supervised DFINE’s learned model had different parameter values, given that its learning procedure is distinct as described above.

### Hyperparameters and learning details

**Extended Data Fig. 8:**
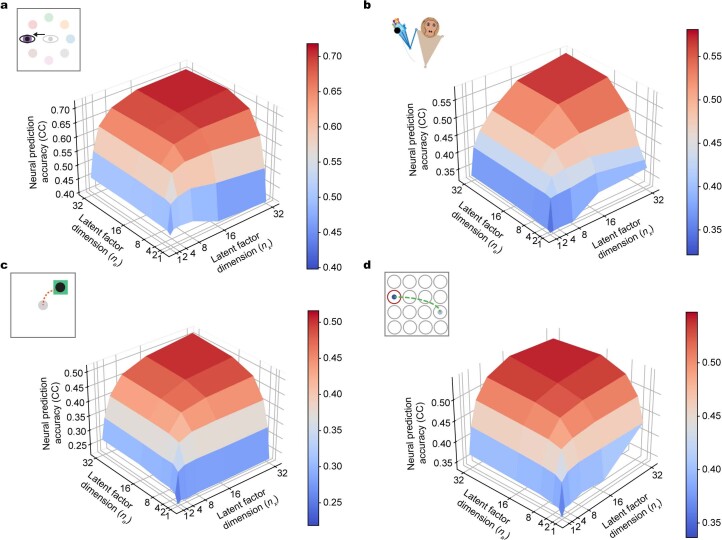
Neural prediction accuracy of DFINE when varying *n*_*x*_ and *n*_*a*_. Neural prediction accuracy for the saccade task (**a**), 3D naturalistic reach-and-grasp task (**b**), 2D random-target reaching task (**c**), and 2D grid reaching task (**d**), for various pairwise values of (*n*_*x*_, *n*_*a*_). This analysis shows that taking *n*_*x*_ = *n*_*a*_ comes at no loss of generality in the neural prediction accuracy as neural prediction accuracy increased the most by increasing *n*_*x*_ and *n*_*a*_ together (Methods). Since both the dynamic and the manifold latent factors can be the bottleneck for information, it intuitively makes sense to increase their dimensionality together for maximum performance and least computational complexity for dimension search.

Given a set of observations, we learned the model parameters by minimizing the cost function in equation (5). We used MLP architectures containing 3 hidden layers, each with 32 units for ${f}_{\theta }(\cdot )$ and ${f}_{\phi }(\cdot )$ in decoder and encoder parts of the model, respectively. The activation function used for the units was set to tanh(⋅). To reduce the computational complexity, we took *n*_*x*_ = *n*_*a*_ in all our analyses throughout the manuscript. Indeed, by characterizing the DFINE neural prediction accuracy with various pairwise (*n*_*x*_, *n*_*a*_) dimensions in Extended Data Fig. 8, we observed that increasing *n*_*x*_ and *n*_*a*_ together improved the neural prediction accuracy the most. It is thus reasonable to increase *n*_*x*_ and *n*_*a*_ together for maximum performance and less computational complexity for the dimension search. We used back propagation through an iterative process of mini-batch gradient descent implemented using ADAM optimizer^127^ to learn the model parameters and we continued the training for 200 epochs. In each iteration, the optimizer computes the cost function *L*(*ψ*, *θ*, *ϕ*), given the current model parameters {*ψ*, *θ*, *ϕ*}, calculates the gradient of the cost with respect to the parameters using back propagation, computes the modified gradient direction and step size, and then takes a step from the current parameter values in the opposite direction of the gradient to minimize the value of the cost. The modification of the gradient direction and the amount of the step size at each iteration were determined by the ADAM optimizer rules^127^. We used 0.02 for the initial learning rate of the ADAM optimizer and continued the training for 200 epochs, where each epoch is a full iteration over the entire training set. We set the maximum horizon for future-step-ahead prediction *K* such that the future predictions cover at least 100 ms into the future, therefore we set *K* = 2–4 to optimize the cost function across various datasets (note that our time step is 50 ms). The following parameters were used for our PyTorch implementation. We used the regularization parameter *λ*_reg_ = 0.002 to prevent overfitting. *λ*_beh_ was set to 20 across all the supervised DFINE models to put emphasis on the improved behaviour prediction accuracy. Note that choices of *λ*_reg_ and *λ*_beh_ are affected by the scale of the input signals, which is affected by *z*-scoring (see cross-validation below).

### Evaluation using 5-fold cross-validation

For all analyses in this work, we performed 5-fold cross-validation, where we divided the data into 5 equal-sized folds, used 4 folds as the training set to learn the model, and left one left-out fold for the test set to evaluate the learned models. Before training and for all methods, we applied *z*-scoring on neural and continuous behaviour signals over time using statistics learned on the training set. Below, we expand on the evaluation metrics used, including behaviour prediction, neural prediction and TDA.

### Behaviour prediction accuracy

#### Saccade task

For any method, we quantified the behaviour prediction accuracy of the inferred latent factor time-series during movement periods by calculating the target classification accuracy using these factors (Fig. 2a). To address the inter-trial variability in the length of movement periods so that the classifier can be applied to latent factors in any trial, we performed an identical preprocessing step for the latent factors from all methods. For this preprocessing, we linearly interpolated the latent factor time-series duration to 400 datapoints and uniformly sampled 10 datapoints (that is, every 40 datapoints after interpolation). After this preprocessing and by flattening the processed factors, we obtained the classification features with dimension equal to 10 × latent factor dimension. For training and test trials, we used these processed features to learn classifiers and perform classification. In addition, to compute the performance given the limited number of trials in the training and test sets, for all methods, we averaged the classification accuracies of all binary classifiers (for each class vs another class) rather than performing an 8-class classification. We used nonlinear support vector machines with Gaussian kernels to perform the binary classification. The width or standard deviation of the Gaussian kernel for each training fold was picked on the basis of inner cross-validation. To assess the target classification accuracy, we used AUC of the receiver’s operating characteristic curve^61^ for all binary classifiers and computed the mean performance for each test cross-validation fold.

#### Motor datasets

Here behaviour variables were continuous. To quantify the behaviour prediction accuracy of the inferred factors by any method, after model training was completed, we learned an MLP regression model from the smoothed latent factors to the observed behaviour variables in the training set. In the test set, we used the learned MLP regression model to get the predicted behaviour variables from the latent factors after they were inferred. We used Pearson’s CC to quantify the behaviour prediction accuracy in each test cross-validation fold.

### Neural prediction accuracy

We quantified the neural prediction accuracy by calculating how accurately models predicted neural observations one-step-ahead into the future from their own past. For the DFINE model, we used ${{\bf{y}}}_{t+1{|t}}={{{f}}}_{\theta }\left({{\bf{a}}}_{t+1{|t}}\right)$ as the one-step-ahead neural prediction. For LDM, neural prediction is given by the classic Kalman predictor^117,125^. However, SAEs do not perform one-step-ahead prediction during the trials/segments as they have a non-causal inference architecture and need to observe all neural observations to infer the latent factors and reconstruct the neural observations (see Discussion). We thus gave SAEs an advantage by allowing them to use all neural observations (instead of just past observations) and thus to perform neural reconstruction instead of one-step-ahead neural prediction. We also compared DFINE’s neural reconstruction accuracy given by ${{\bf{y}}}_{{t|T}}={{{f}}}_{\theta }\left({{\bf{a}}}_{{t|T}}\right)$ to SAE’s neural reconstruction accuracy in Supplementary Fig. 2, with similar conclusions. We used Pearson’s CC to quantify the neural prediction accuracy in each test cross-validation fold.

### TDA metrics

To quantify how robustly models identified the latent manifold structure in single trials, we applied TDA^62^ on smoothed latent factors. TDA uses persistent homology^62^ to find multidimensional holes (for example, 1D hole is a ring, 2D hole is a 2D void) in data by growing the radius of ***ϵ***-balls around datapoints, which are connected when they touch. TDA finds the manifold type by counting the number of persistent multidimensional holes in the data manifold. For example, a torus has one 2D hole and two 1D holes^62^. We ran TDA on smoothed single-trial latent factors of the learned models in the cross-validation test set and assessed the most persistent hole’s birth and length. The most persistent hole is the hole that lasts the longest. The birth happens at the ***ϵ*** value at which the hole appears (smaller values correspond to earlier births) and the length is the ***ϵ*** interval for which the hole lasts. To assess robustness in single trials, we asked for which model TDA finds holes that are born earlier and last longer, that is, are more persistent: the sooner a hole is born (at shorter radius) and the longer it lasts (at longer radius), the more prevalent/robust it is in the latent factors extracted. To take into account scaling differences in the latent space of each method, we *z*-scored single-trial latent factors in each dimension before running TDA. To visualize the TDA results, for the most persistent holes, we plotted their lengths vs births. On this plot, the optimal location is the top left area of the plot indicating earlier births and longer lengths. To aggregate the results in each test cross-validation fold, we averaged the birth and length values of TDA for single-trial latent factors in that test fold.

### Implementation details for benchmark methods

#### SAE

For SAE, we used the architecture named LFADS^5^, which is a common benchmark nonlinear model of neural population activity^13,49,52^. LFADS is an RNN-based variational SAE^5^. LFADS takes fixed-length segments of data as input and encodes each segment into a bottleneck latent factor, which serves as the initial condition for the decoder’s network (that is, generator). Given this initial condition, the generator RNN propagates the latent states, generates a factor time-series, which then reconstructs a smoothed copy of the input data segment. We trained LFADS using the publicly available source code^5^ and using hyperparameters in row 2 of Supplementary Table 1 in ref. ^5^ because the number of trials in the dataset associated with row 2 was closest to our datasets. The dimensionality of the generator RNN’s latent state in LFADS represents its dynamical memory and the number of values used at any given time step to generate the dynamics/states of the next time step, thus representing its dynamics state dimension^13^. We kept the generator RNN’s latent state dimension (and thus the initial condition latent factor dimension) high enough and set it to 64. This gives an advantage to LFADS in terms of modelling the dynamics because it gives LFADS a higher dynamics state dimension of 64 compared with DFINE that has this dimension at 16. We used a Gaussian observation distribution to train LFADS so that, similar to DFINE, it can be applied to all neural modalities considered here, whether Gaussian-smoothed firing rates or LFP. We used the developers’ LFADS implementation, which provides the option for the Gaussian observation distribution. We used the LFADS factor time-series as the latent factors of LFADS in our analyses/comparisons as was done in ref. ^5^.

LFADS can only be trained on 3D data tensors (trial × time × observation dimension) given its SAE structure. Thus, for its model training and inference, we split the continuous data into smaller 1 s segments to create 3D data tensors in both training and test sets^13,110^. We then trained the LFADS model with the training set and performed inference in each 1 s segment of data. We finally got the smoothed latent factors for the full duration of training and test sets by concatenating the inferred latent factors across segments. Similar to ref. ^5^, we ran the inference 50 times, as LFADS has a variational autoencoder format^5^, and averaged the inferred latent factors from these 50 realizations.

#### fLDS

We also compared DFINE against fLDS which provides another competitive nonlinear dynamical model of neural population activity^50^. In fLDS, the latent factors **x**_*t*_ are modelled with a linear state equation and the observations are modelled with a nonlinear function of the latent factors ${{\bf{y}}}_{t}={{g}}\left({{\bf{x}}}_{t}\right)+{{\bf{v}}}_{t}$. This is different from the DFINE model architecture that has two sets of latent factors, including the extra noisy manifold latent factor. Further, the nonlinearity applied in DFINE on the dynamic latent factors is stochastic (see ${{{f}}}_{\theta }\left(\cdot \right)$ with respect to **x**_*t*_ in section ‘DFINE model’ above), while in fLDS, the nonlinearity ${{g}}\left(\cdot \right)$ with respect to **x**_*t*_ is deterministic^50^. In terms of inference, fLDS performs non-causal (smoothing) inference and does not directly address missing neural observations. In terms of learning, fLDS optimizes the ELBO cost, while DFINE optimizes the future-step-ahead prediction cost and can do so because of its flexible inference capability to efficiently and recursively compute this cost during training (see also Supplementary Table 1). We used the hyperparameters mentioned in ref. ^50^ and in the developers’ GitHub repository (https://github.com/earcher/vilds) to learn fLDS on our data. Please note that similar to all other benchmark methods, the observation distribution was set to be Gaussian in fLDS using the developers’ code. Since fLDS only allows for smoothing inference, and to get the one-step-ahead prediction estimates at each time *t*, we performed smoothing inference (from **y**_1:*t*_) to get ${{\bf{x}}}_{{{t}}|{{t}}}$ and obtain the one-step-ahead prediction of the observations as ${{g}}\left({{A}}{{\bf{x}}}_{{{t}}|{{t}}}\right).$ Throughout the paper, **x**_*t*_ in the fLDS model was used as its latent factors.

#### LDM

Similar to our previous work^13^, we trained LDM models using a publicly available Python package that performs subspace identification^13^. Throughout the paper, we used the states of the LDM model as the latent factors. The latent factors were inferred using Kalman filtering and smoothing.

### Nonlinear manifold simulations

We validated the DFINE model on numerical simulations first. Since previous studies on neural population activity have shown evidence for rotatory dynamics and ring-like/Toroidal manifolds^7,11,16,69^, we simulated the nonlinear manifold structure using three different manifold types to show generality: ring-like, Torus and Swiss-roll manifolds. We simulated nonlinear trajectories over these manifolds by first generating a driven walk using an LDM on the manifold local coordinates and then embedding these coordinates in 3D cartesian space using the manifold equations (see Supplementary Note 1). To get the neural observations, we generated 40D output signals by first applying a random output emission matrix (of size 40 × 3) on the 3D trajectories and then adding additive white Gaussian noise to the 40D signal to realize noisy observations. Without loss of generality and only for illustration purposes, we kept the first 3 dimensions of the observations the same as the 3D trajectories (that is, used identity output submatrix for the first 3 dimensions) so that we could illustrate the first 3 dimensions for our 3D visualizations without any linear distortions. We emphasize that all quantifications were calculated with 40D observations (see Supplementary Note 1 for more details). We simulated 30 different sessions (10 from each manifold type), where we randomly picked the white Gaussian noise standard deviations. In each session, we simulated 250 trials, each containing 200 time steps. Our validations were all based on 5-fold cross-validation on these trials.

In addition to visualization, we quantified the success of learning with the convergence of one-step-ahead neural prediction error in the learned models to that of the true model. We calculated the one-step-ahead prediction error using NRMSE; this normalization allowed for pooling of the results across sessions, given potential scaling differences across various simulated sessions. Given a 1D signal ${{y}}_{t}$ and its prediction ${\hat{y}}_{t}$, NRMSE was calculated as:

7 $${\rm{NRMSE}}=\frac{\sqrt{\sum _{t}{\left({y}_{t}-{\hat{y}}_{t}\right)}^{2}}}{\sqrt{\sum _{t}{\left({y}_{t}-\bar{y}\right)}^{2}}},$$

where $\bar{y}$ is the mean of the signal. We calculated the NRMSE for each dimension and report the mean across dimensions for the 40D observations in our case. To calculate the one-step-ahead prediction error, we computed the NRMSE between the true observations (${\bf{y}}_{t}$) and their predictions one step into the future (${\hat{{\bf{y}}}}_{{t|t}-1}$). For the true models, we calculated the one-step-ahead predictions using unscented Kalman filtering (UKF)^128^ as the true manifolds have nonlinear embeddings that we can write analytically for the true model and write a UKF for.

### Lorenz attractor system simulations

We performed a simulation analysis using a well-known system with nonlinear temporal dynamics, the stochastic Lorenz attractor system. This system is commonly used to test nonlinear latent state methods^5,52^. The stochastic Lorenz attractor system is a set of nonlinear dynamical equations with 3 dynamic latent factors:

8 $$\begin{array}{rcl}{{{dx}}}_{1}&=&{\sigma} \left({{{x}}}_{2}-{{{x}}}_{1}\right){{dt}}+{{{q}}}_{1}\\ {{{dx}}}_{2}&=&(\rho {{{x}}}_{1}-{{{x}}}_{1}{{{x}}}_{3}-{{{x}}}_{2}){{dt}}+{{{q}}}_{2}\\ {{{dx}}}_{3}&=&({{{x}}}_{1}{{{x}}}_{2}-\beta {{{x}}}_{3}){{dt}}+{{{q}}}_{3},\end{array}$$

where *x*_1_, *x*_2_ and *x*_3_ are the latent factors, and *q*_1_, *q*_2_ and *q*_3_ are the white Gaussian noises for each latent factor dimension. The infinitesimal change in variables is denoted by *d*. We used *σ* = 10, *ρ* = 28 and $\beta =\frac{8}{3}$ for Lorenz model parameters, similar to a previous work^5^, and Euler integration with *dt* = 0.01 to simulate the system dynamics. Similar to our manifold simulations, we generated 40D output signals by first applying a random output emission matrix (of size 40 × 3) on the 3D Lorenz trajectories and then adding additive white Gaussian noise (variance = 1) to the 40D signal to realize noisy observations. We swept the dynamics noise variable magnitude (variance of *q*_*i*_) from 10^−4^ to 1 and for each value, we generated 20 simulated sessions. We simulated 750 trials for 200 time steps for each session, and we performed 5-fold cross-validation to assess the results. The initial condition for each trial of the stochastic Lorenz simulation was obtained after 500 burn-in steps starting from a random latent factor sampled from a normal distribution^5^.

We used DFINE and SAE models with latent factor dimension of 3 to learn the stochastic Lorenz attractor system’s dynamics. For SAE, we used the LFADS architecture with the hyperparameter set corresponding to the Lorenz attractor simulations reported in Supplementary Table 1 of ref. ^5^. Similar to ref. ^5^ and for each method, we learned the linear projection from the inferred latent factors to the actual Lorenz latent factors, used the linear projection to reconstruct the Lorenz latent factors in the test set and then quantified the Lorenz latent factor reconstruction accuracy with CC.

### Inference analyses with missing observations

We assessed/visualized the inference in the presence of missing observations across various observed datapoint ratios. Observed datapoint ratio, denoted by *ρ*, quantifies the ratio between the number of observed datapoints versus the total number of datapoints. We trained both the DFINE and SAE models on fully observed training sets and then tested their inference on test sets with randomly missing observations. For simulation analyses, we varied *ρ* from 0.05 to 1 by randomly dropping datapoints in each test trial, inferred the latent factors in the presence of missing/dropped observations and then quantified the error between the true and reconstructed manifold trajectories via filtering/smoothing. For motor datasets, we introduced missing observations by randomly and uniformly dropping neural observations with various observed datapoint ratios (*ρ* ranging from 0.2 to 1). Since the motor datasets contained continuous recordings, and to make sure that we dropped datapoints uniformly throughout the duration of the time-series, we randomly dropped (1–*ρ*) × 100 datapoints in every 100 time steps of the neural observation time-series.

Using the learned DFINE models, in the test set, we inferred the latent factors at all time steps even though observations were missing at some random time steps. From these latent factors, the behaviour variables were predicted in the test set using the learned MLP models and thus the cross-validated behaviour prediction accuracy was computed. Note that even though observations were missing at random time steps, the latent factors and thus behaviour variables were inferred at all time steps. In a control analysis, we also performed inference with LDM and SAE in the presence of missing observations as described above. For LDM, the latent factors were inferred with Kalman filtering/smoothing where at the time of missing observations, the latent estimate in the forward pass was obtained by the Kalman predictor. For SAEs, we used two different imputation techniques since SAE’s encoder RNN is designed to take inputs at every time step: (1) impute the missing observations with zeros as done previously^53,54^ and (2) impute the missing observations with the average of the last and the next/future available observations. Given the SAE models that were trained on fully observed training sets, we inferred the latent factors in the test sets with missing observations, predicted behaviour variables with learned MLP models and computed the cross-validated behaviour prediction accuracy.

### Statistical analysis

For all analyses in this work, significance was declared if *P* < 0.05. All statistical tests were performed with non-parametric Wilcoxon signed-rank tests.

### Reporting summary

Further information on research design is available in the Nature Portfolio Reporting Summary linked to this article.

## Supplementary information

Supplementary Information Supplementary Figures, Tables, Notes and References.

Reporting Summary

## Data Availability

The main data supporting the results in this study are available within the paper and its Supplementary Information. Two of the datasets used for this work are publicly available from the Miller and Sabes labs at the following links: dataset 3 at 10.6080/K0FT8J72 and dataset 4 at 10.5281/zenodo.3854034. The other two datasets used to support the results are too large to be publicly shared, yet they are available for research purposes from the corresponding author on reasonable request.

## Code availability

The custom computer code of DFINE is available at https://github.com/ShanechiLab/torchDFINE.
