# Author Correction: Dynamical flexible inference of nonlinear latent factors and structures in neural population activity

**DOI:** 10.1038/s41551-025-01345-4

**Published:** 2025-01-15

**Authors:** Hamidreza Abbaspourazad, Eray Erturk, Bijan Pesaran, Maryam M. Shanechi

**Affiliations:** 1 Ming Hsieh Department of Electrical and Computer Engineering, Viterbi School of Engineering, University of Southern California, Los Angeles, CA USA; 2 Departments of Neurosurgery, Neuroscience, and Bioengineering, University of Pennsylvania, Philadelphia, PA USA; 3 Thomas Lord Department of Computer Science, Alfred E. Mann Department of Biomedical Engineering, Neuroscience Graduate Program, University of Southern California, Los Angeles, CA USA

**Keywords:** Computational neuroscience, Biomedical engineering, Computational science, Motor control

Correction to: *Nature Biomedical Engineering* 10.1038/s41551-023-01106-1, published online 11 December 2023.

This paper was originally published under standard Springer Nature license (© The Author(s), under exclusive licence to Springer Nature Limited). It is now available as an open-access paper under a Creative Commons Attribution 4.0 International license, © The Author(s). The error has been corrected in the online version of the article.

