# Supplementary material for: Dynamical flexible inference of nonlinear latent factors and structures in neural population activity
Source: Nat Biomed Eng. 2023 Dec 11;8(1):85–108. doi: 10.1038/s41551-023-01106-1 (PMC11735406; doi:10.1038/s41551-023-01106-1)
Supplement: Supplementary file 1 — Supplementary Figures, Tables, Notes and References. [file 41551_2023_1106_MOESM1_ESM.pdf]

nature biomedical engineering

Article https://doi.org/10.1038/s41551-023-01106-1

Dynamical flexible inference of nonlinear latent factors and structures in neural population activity

In the format provided by the authors and unedited

Contents

Supplementary Fig. 1 | Neural prediction accuracy as a function of the latent factor dimension for all datasets and methods.
Supplementary Fig. 2 | DFINE's neural reconstruction accuracy with smoothing is also better than that of SAE.
Supplementary Fig. 3 | Example latent factor trajectories for the motor datasets.
Supplementary Fig. 4 | DFINE more robustly extracts the ring-like manifold structure in single-trials during the preparation period of the saccade task.
Supplementary Fig. 5 | Supervised DFINE had lower neural prediction accuracy compared with DFINE as expected, because the former optimizes for both neural and behaviour prediction simultaneously rather than just for neural prediction.

Supplementary Table 1 | Total training and total inference runtimes in seconds.

Supplementary Note 1 | Details of numerical simulations.

Supplementary References

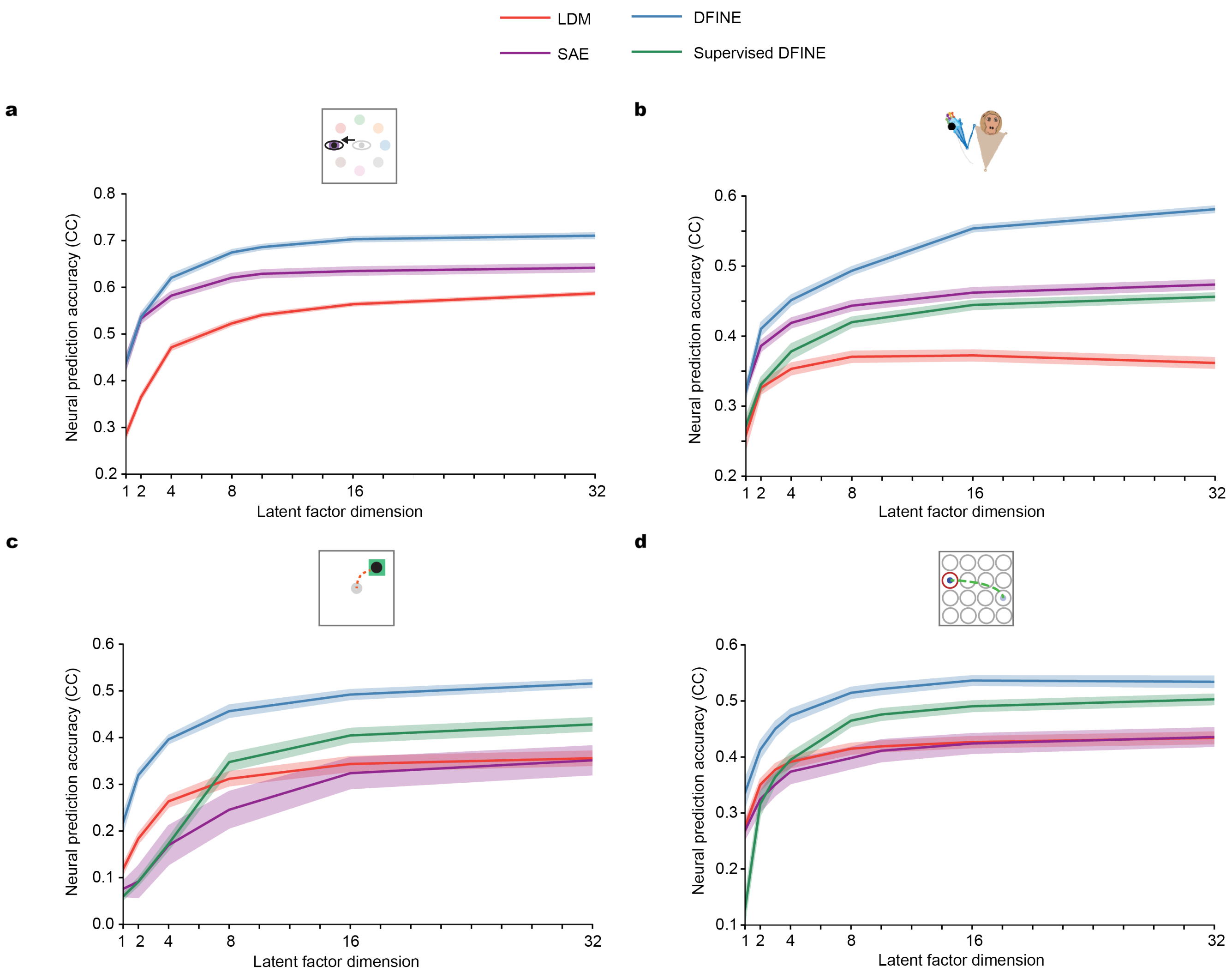

Supplementary Fig. 1 | Neural prediction accuracy as a function of the latent factor dimension for all datasets and methods. Solid lines show the mean neural prediction accuracy across sessions and cross validation folds for the saccade task (observation dimension $n_y = 32$) **(a)**, 3D naturalistic reach-and-grasp task (observation dimension $n_y = 30$) **(b)**, 2D random-target reaching task (observation dimension $n_y = 46 - 57$) **(c)**, and 2D grid reaching task (observation dimension $n_y = 30$) **(d)**. While not displayed here, we performed a sanity check and confirmed that as the factor dimensions increased and became substantially higher than the neural observation dimension (e.g., latent factor dimension of 128 for the three motor datasets and 64 for the saccade dataset), overfitting happened in training the DFINE model and prediction accuracy went down in the test set as expected. The shaded areas represent the 95% confidence bound. For SAE, the dimension shown is the factor dimension and the dynamic dimension (generator RNN's latent state dimension and initial condition dimension) is always 64.

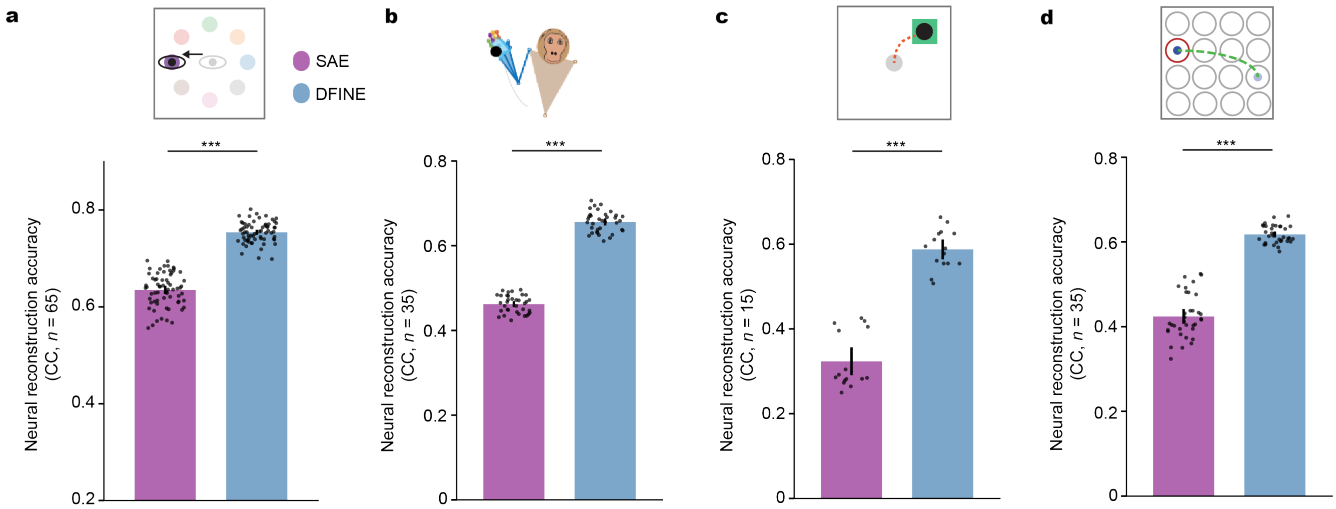

Supplementary Fig. 2 | DFINE's neural reconstruction accuracy with smoothing is also better than that of SAE. Figure convention is as in **Fig. 4**. The neural reconstruction accuracy with smoothing is shown for the saccade task **(a)**, 3D naturalistic reach-and-grasp task **(b)**, 2D random-target reaching task **(c)**, and 2D grid reaching task **(d)**.

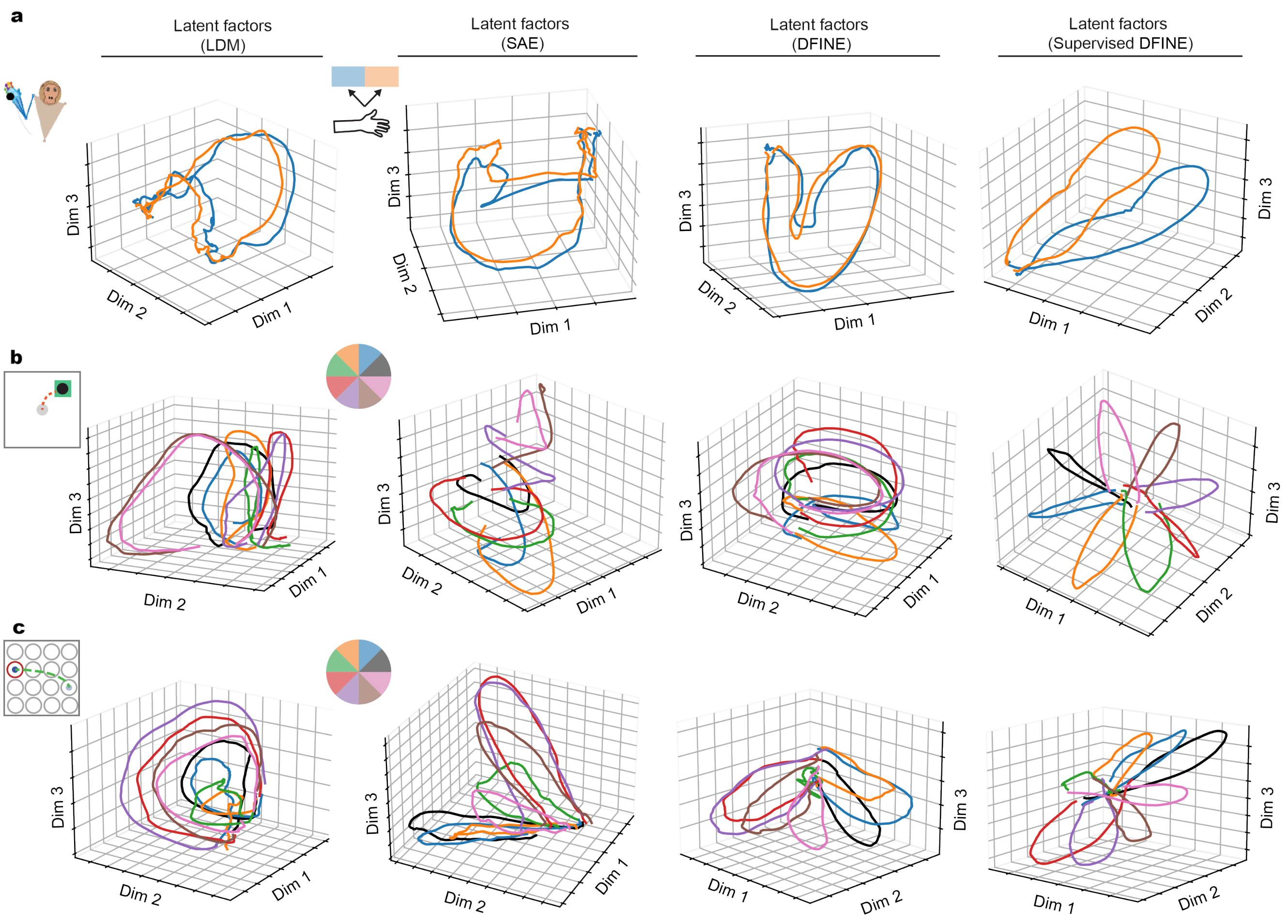

Supplementary Fig. 3 | Example latent factor trajectories for the motor datasets. Figure convention for the conditions is as in **Fig. 6**. The condition-average latent factor trajectories are shown for all methods in the 3D naturalistic reach-and-grasp task **(a)**, 2D random-target reaching task **(b)**, and 2D grid reaching task **(c)**. We observed a ring-like manifold structure during the movement periods and DFINE more robustly identified this ring-like structure in single-trials as evidenced by the TDA results in **Figs. 4** and **5**. DFINE's ring-like manifold structure was more predictive of neural activity (**Figs. 4** and **5**), suggesting that it provided a good description of data.

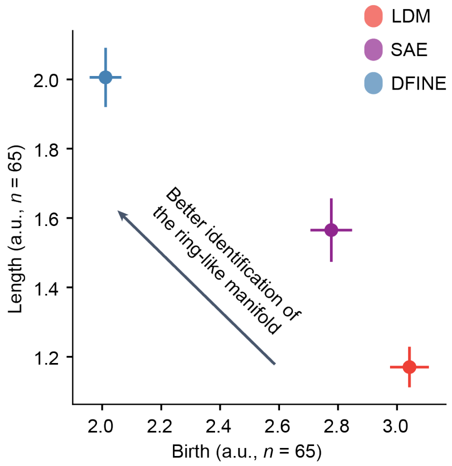

Supplementary Fig. 4 | DFINE more robustly extracts the ring-like manifold structure in single-trials during the preparation period of the saccade task. TDA analysis on single-trial latent factors during the preparation period is shown. TDA's most persistent 1D hole had a significantly earlier birth and lasted significantly longer for DFINE compared to LDM and SAE ($P < 5 \times 10^{-4}$, one-sided Wilcoxon signed-rank test, $n = 65$). Figure convention is the same as **Fig. 4e**. Example condition-average and single-trial latent factor trajectories during the preparation period are shown in **Fig. 4a**.

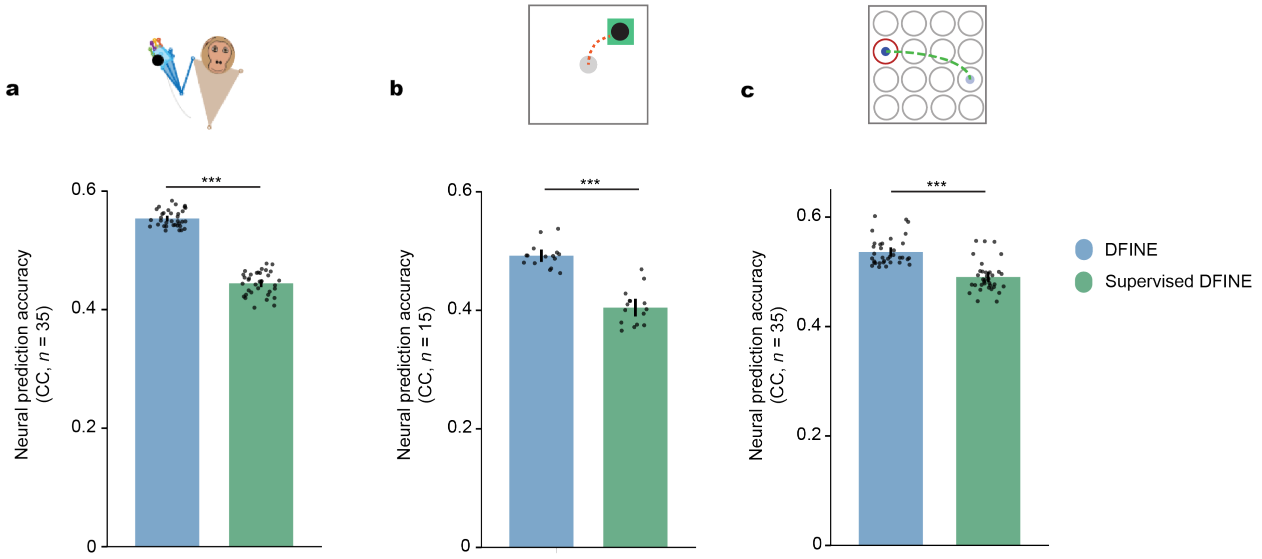

Supplementary Fig. 5 | Supervised DFINE had lower neural prediction accuracy compared with DFINE as expected, because the former optimizes for both neural and behaviour prediction simultaneously rather than just for neural prediction. Figure convention is as in **Fig. 6b**. Neural prediction accuracies are shown for the 3D naturalistic reach-and-grasp task **(a)**, 2D random-target reaching task **(b)**, and 2D grid reaching task **(c)**.

Supplementary Table 1 | Total training and total inference runtimes in seconds. Runtimes are provided for all methods in this study for a toy dataset with 250 trials where each trial has 200 time-steps. The data was trained and tested in a 5-fold cross-validation manner providing 5 runtime values for each case. Reported numbers are the $\mathrm{mean} \pm \mathrm{std}$ across the 5 folds. Inference runtimes are the total runtime for inferring all time-steps across all trials within the train/test sets. Models were trained on xeon-2650v2 CPU with CentOS Linux 7 operating system. We used the developer's Python code and deep learning framework in each instance, the details of which are provided in their codebase and prior papers[1–3] (**Methods**). For LDM, there is no iterative optimization like the other methods, so the total and per-epoch training runtimes are identical.

Method Name	Training runtime (total, sec)	Training runtime (per epoch, sec)	Latent factor inference runtime (train, sec)	Latent factor inference runtime (test, sec)
**LDM**	13.63 ± 0.17	NA	1.45 ± 0.03	0.36 ± 0.01
**SAE**	6224.73 ± 329.55	4.44 ± 0.18	291.64 ± 24.40	72.04 ± 6.29
**fLDS**	11573.04 ± 129.80	57.87 ± 0.65	11.03 ± 0.18	2.73 ± 0.03
**DFINE**	723.94 ± 16.55	3.62 ± 0.08	2.21 ± 0.21	0.51 ± 0.07

Supplementary Note 1 | Details of numerical simulations

We simulate 3 different manifold types including ring-like, Torus, and Swiss roll manifolds. Here, we expand on the numerical simulations.

Manifold equations

For each manifold type, we first get the 3-dimensional (3D) manifold embeddings, which specify how the manifold is embedded in 3D Cartesian space and are used as visualizations (**Supplementary Note Fig. 1**). We then transform these embeddings using a random output emission matrix to a 40D space to get the neural observations (see next section). To get the 3D manifold embeddings, each manifold type has its own equation, which we expand on in the following sections. Below, we denote the 3 dimensions of the 3D embeddings as e_1, e_2 and e_3.

Ring-like manifold: We generate the ring-like manifold embeddings from the 1D ring manifold coordinate (d_θ), which is an angle between 0-2π, using the following equations (**Supplementary Note Fig. 1a**):

$$\begin{aligned} e_1 &= \cos(\mathrm{d}_\theta), \\ e_2 &= \sin(2d_\theta), \\ e_3 &= \sin(d_\theta). \end{aligned} \tag{1}$$

Torus manifold: We generate the Torus manifold embeddings from the 2D Torus manifold coordinates d_r and d_R (**Supplementary Note Fig. 1b**). Here d_r is the coordinate – angle between 0-2π – for the minor circle, which is the inner circle representing the Torus's tube. Also, d_R is the coordinate – angle between 0-2π – for the major circle, which is the outer circle on which the Torus' tube evolves. Further, R and r are the radius values for the major and minor circles. We get the 3D manifold embeddings as:

$$\begin{aligned} e_1 &= \big(R + r\cos(d_R)\big)\cos(d_r), \\ e_2 &= \big(R + r\cos(d_R)\big)\sin(d_r), \\ e_3 &= r\sin(d_R). \end{aligned} \tag{2}$$

Without loss of generality, we use $R = 4$ and $r = 1.5$ for the major and minor radii, respectively.

Swiss roll manifold: The below equation generates the 3D Swiss roll manifold embeddings from the 2D Swiss-roll coordinates d_r and d_h, which are its circular and height coordinates, respectively (**Supplementary Note Fig. 1c**):

$$\begin{aligned} e_1 &= 0.5 \times d_r\cos(d_r), \\ e_2 &= d_h, \\ e_3 &= 0.5 \times d_r\sin(d_r). \end{aligned} \tag{3}$$

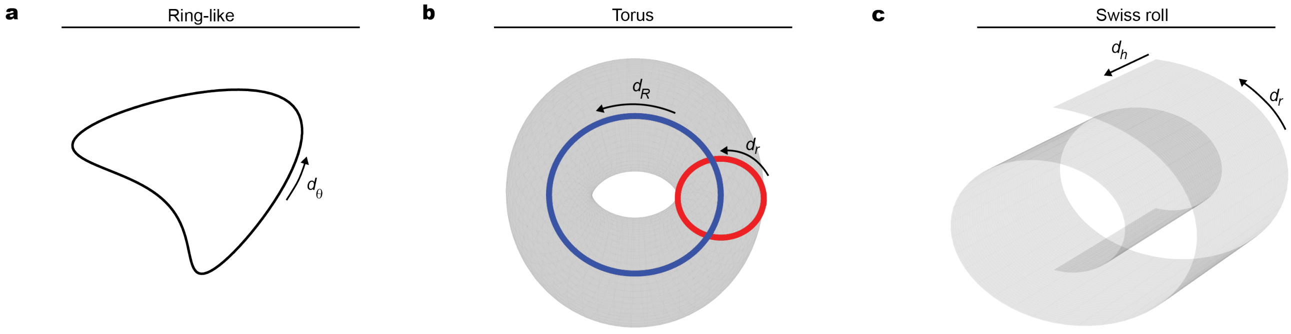

Supplementary Note Fig. 1 | Visualizations of the manifolds. 3D manifold embeddings are shown for ring-like **(a)**, Torus **(b)**, and Swiss roll **(c)** manifolds.

Generating neural trajectories over manifolds

We denote the vector manifold coordinates of the trajectories at each time step by $\mathbf{d}_t$. Thus $\mathbf{d}_t$ is $[d_\theta]$, $[d_r; d_R]$ and $[d_r; d_h]$ for ring, Torus and Swiss roll manifolds, respectively. We generate trajectories over the manifolds by first generating a walk on the manifold's local coordinate space with a linear dynamical equation:

$$\mathbf{d}_{t+1} = A_d \mathbf{d}_t + \mathbf{b} + \mathbf{q}_t, \tag{4}$$

where A_d is the diagonal state transition matrix with eigenvalues of 0.99, $\mathbf{b}$ is the input term to drive the trajectory at each time-step (set as 0.2), and $\mathbf{q}_t$ is the white Gaussian noise with covariance Q. The standard deviation of $\mathbf{q}_t$ is randomly chosen between $[0.01, 0.1]$. After generating the trajectories from equation (4), we embed the manifold coordinate vector $\mathbf{d}_t$ within 3D Cartesian space to get the embeddings $\mathbf{e}_t$ with equations (1)-(3) and we finally get the neural observations from:

$$\boldsymbol{y}_t = T\mathbf{e}_t + \mathbf{o}_t, \tag{5}$$

where T is the output emission matrix and $\mathbf{o}_t$ is a white Gaussian noise with covariance matrix O. The matrix $T \in \mathbb{R}^{40\times3}$ has its first 3 rows chosen as $[1,0,\dots,0], [0,1,0,\dots,0], [0,0,1,0,\dots,0]$ to form an identity transformation for the first 3 dimensions of the manifold (for visualization purposes only), and the rest of the 37 rows are randomly chosen with elements between $[-5,5]$. The standard deviation of $\mathbf{o}_t$ is randomly chosen between $[5,25]$.

Supplementary References

1. Pandarinath, C. *et al.* Inferring single-trial neural population dynamics using sequential auto-encoders. *Nat. Methods* **15**, 805–815 (2018).
2. Sani, O. G., Abbaspourazad, H., Wong, Y. T., Pesaran, B. & Shanechi, M. M. Modeling behaviorally relevant neural dynamics enabled by preferential subspace identification. *Nat. Neurosci.* **24**, 140–149 (2021).
3. Gao, Y., Archer, E. W., Paninski, L. & Cunningham, J. P. Linear dynamical neural population models through nonlinear embeddings. *Adv. Neural Inf. Process. Syst.* **29**, 163–171 (2016).
